# A First-Principles Thermodynamic Model for the Ba–Zr–S System in Equilibrium with Sulfur Vapor

**DOI:** 10.1021/acsaem.3c03208

**Published:** 2024-04-05

**Authors:** Prakriti Kayastha, Giulia Longo, Lucy D. Whalley

**Affiliations:** Department of Mathematics, Physics and Electrical Engineering, Northumbria University, Newcastle-upon-Tyne NE1 8QH, United Kingdom

**Keywords:** perovskite, solid-state, simulation, first-principles, thermodynamics, sulfur

## Abstract

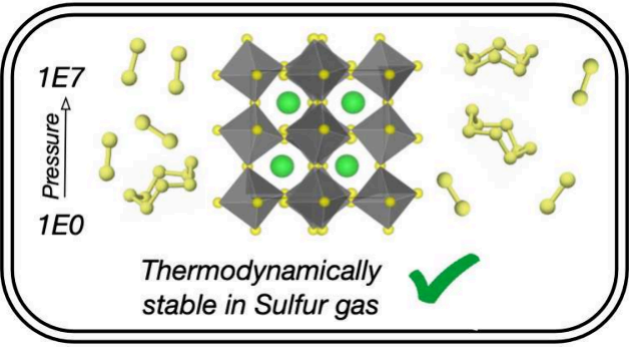

The chalcogenide perovskite BaZrS_3_ has strong visible light absorption and high chemical stability, is nontoxic, and is made from earth-abundant elements. As such, it is a promising candidate material for application in optoelectronic technologies. However, the synthesis of BaZrS_3_ thin-films for characterization and device integration remains a challenge. Here, we use density functional theory and lattice dynamics to calculate the vibrational properties of elemental, binary, and ternary materials in the Ba–Zr–S system. This is used to build a thermodynamic model for the stability of BaZrS_3_, BaS_*x*_, and ZrS_*x*_ in equilibrium with sulfur gas across a range of temperatures and sulfur partial pressures. We highlight that reaction thermodynamics are highly sensitive to sulfur allotropes and the extent of allotrope mixing. We use our model to predict the synthesis conditions in which BaZrS_3_ and the intermediate binary compounds can form. At a moderate temperature of 500 °C, we find that BaS_3_, associated with fast reaction kinetics, is stable at pressures above 3 × 10^5^ Pa. We also find that BaZrS_3_ is stable against decomposition into sulfur-rich binaries up to at least 1 × 10^7^ Pa. Our work provides insights into the chemistry of this promising material and suggests the experimental conditions required for the successful synthesis of BaZrS_3_.

## Introduction

Chalcogenide perovskites are a class of perovskite materials that have recently gained significant attention as lead-free alternatives for photovoltaic (PV) applications.^1−3^ They are environmentally stable and exhibit a range of desirable properties for optoelectronic applications, including defect tolerance,^4^ strong dielectric screening,^5^ and high charge carrier mobility.^6^ BaZrS_3_ is the most studied material in this class due to its stability and high absorption coefficient.^7,8^ A wide bandgap in the range of 1.8 to 2.0 eV^9^ makes it a suitable material for integration as a top cell absorber in Si–perovskite tandem applications.^10^ In addition, the bandgap can be reduced through mixing on the Ba site,^11^ Zr site,^11−13^ or chalcogenide site,^14,15^ leading to the possibility of a single junction BaZrS_3_ solar cell.

Although BaZrS_3_ exhibits promising physical and chemical properties, a scalable synthesis method which avoids high temperatures and/or long reaction times remains an open challenge. During solid state synthesis, there are kinetic mass transport limitations when annealing the stable binary reactants; highly crystalline films are deposited at temperatures in the range of 800 to 1100 °C.^10,16^ While the high kinetic barriers in chalcogenide perovskites might contribute to their thermal and chemical stability, the resulting high synthesis temperatures preclude thin-film growth on a photovoltaic device stack.

To overcome kinetic limitations, several groups are exploring the use of a barium polysulfide liquid BaS_*x*_, *x* > 3, as a reaction intermediate.^1,17−19^ This has lowered the temperature required for perovskite synthesis through the formation of a liquid flux of sulfur-rich barium polysulfide at around 550 °C.^20,21^ This approach allows for BaZrS_3_ synthesis using heat treatment at moderate temperatures in a sulfur-containing atmosphere.

Despite this recent progress in BaZrS_3_ synthesis at moderate temperatures, the underlying mechanisms and experimental conditions required for perovskite formation are not well understood. Initial experimental results have suggested there is a window of sulfur partial pressure within which the formation of BaZrS_3_ is favored.^1^ However, it is unclear whether this window is determined by kinetic factors limiting the rate of perovskite formation^19^ or the thermodynamic instability of BaZrS_3_ with respect to sulfur-rich binary materials BaS_3_ and ZrS_3_.

A complicating factor is that in the gas phase sulfur forms a range of S_*n*_ allotropes at relative ratios which are highly sensitive to temperature and pressure,^22^ and this mixing can have a significant impact on predicted formation energies in the experimental conditions used for perovskite synthesis.^23^ However, the commonly made assumption is that the sulfur gas consists of a single species (most often S_2_ or S_8_), which is determined by the temperature of the sulfur source. Furthermore, experimental characterization and control of the sulfur allotrope(s) formed is highly challenging.

**Figure 1:**
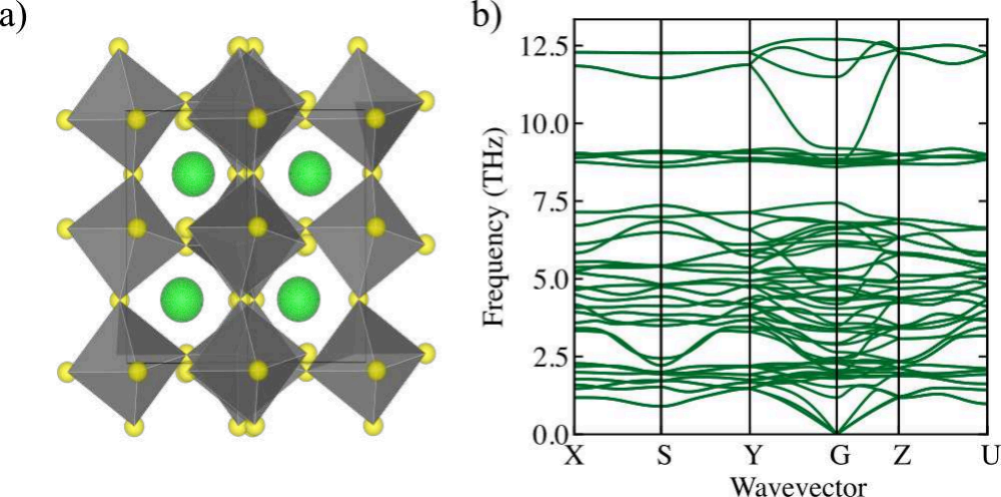
(a) Relaxed crystal structures of BaZrS_3_ in the *Pnma* orthorhombic perovskite phase. Ba atoms are green, S atoms are yellow, and the Zr atoms, which lie in the center of each octahedra (shaded gray), are not shown. (b) Harmonic phonon bandstructure of BaZrS_3_ showing positive phonon modes across the Brillouin zone.

In this work, we build a thermodynamic model for elemental, binary, and perovskite materials in the Ba–Zr–S system. We perform density functional theory and harmonic phonon calculations (Figure 1) to predict Gibbs free energies as a function of temperature and pressure. Combining this with published experimental data^24^ and a parametrized model^23^ for sulfur vapor allows us to incorporate the effects of sulfur partial pressure for the single-species S_2_ and S_8_ allotropes, in addition to an equilibrium sulfur gas with allotrope mixing. Through this we demonstrate the impact of sulfur species on reaction thermodynamics and predict the synthesis conditions required for BaZrS_3_ formation. The first-principles data and material-agnostic postprocessing code underlying this model are freely available online.^25−27^

In our analysis, we consider reaction pathways between the perovskite and competing binary and elemental phases. Ruddlesden–Popper (RP) phases are reported to form at high temperatures^28−31^ or for kinetically limited Zr precursors.^19^ RP phases are not examined here as we focus our attention on perovskite formation at moderate temperatures.

## Methodology

### Classical Thermodynamics

We use classical thermodynamics to evaluate the stability of materials through the balance of free energies. The feasibility of a spontaneous reaction at temperature *T* and total pressure *P* is determined by the change in Gibbs free energy Δ*G*:
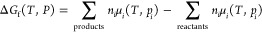
1where *n*_*i*_ is the absolute change in stoichiometry, *μ*_*i*_ is the chemical potential, and *p*_*i*_ is the partial pressure of each product or reactant. For reactions with Δ*G* < 0, the reaction is energy releasing and thermodynamically favorable. We note that meeting this condition is not necessarily sufficient for forming a given product over a reasonable time scale; kinetic factors may inhibit the reaction.

We assume that solids are incompressible and consider vibrational contributions to entropy only, giving the following expression for Gibbs free energy at finite temperature and pressure:^32^
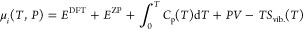
2Density functional theory (DFT) is used to calculate the total electronic energy *E*^DFT^ and equilibrium volume *V* for a static crystal at 0 Pa. We use first-principles lattice dynamics to calculate the zero point energy *E*^ZP^, vibrational entropy *S*_vib._(*T*), and heat capacity *C*_p_(*T*). Note that *C*_p_ = *C*_v_ for incompressible solids.

We assume that S_2_ and S_8_ follow the ideal gas law, giving:^32^
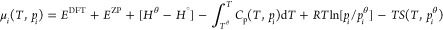
3The NIST-JANAF thermochemical data tables provide experimental data for the standard enthalpy [*H*^θ^ – *H*^0^], heat capacity *C*_p_(*T*, *p*_*i*_) and entropy *S*(*T*, *p*_*i*_^θ^).^24^ θ denotes the reference state used in the tables (*T* = 298 K, *P* = 1 × 10^5^ Pa). *E*^ZP^ values are taken from the NIST Computational Chemistry Comparison and Benchmark Database.^33^ We reference the *E*_DFT_ value for S_2_ against *E*_DFT_ for S_8_ using the energy difference employed to construct the mixed allotrope model in ref (23). This energy difference is calculated by a hybrid PBE0 functional^34^ and is in close agreement with reference data.^24^ Calculations with the SCAN,^35^ PBEsol,^36^ and HSE06^37^ functionals overestimate the energy difference, leading to a coexistence curve that is significantly too high in temperature (see Figures S28 and S29). To compare calculated *μ* values across sulfur allotropes, we report *μ* on a per-atom basis, i.e., *μ*_S_8__/8 for S_8_ and *μ*_S_2__/2 for S_2_.

To account for allotrope mixing, we applied a published first-principles model for the chemical potential of sulfur gas.^23^ This model considers 13 low-energy allotropes of sulfur (S_2_–S_8_) and is parametrized using first-principles lattice dynamics with the hybrid PBE0 functional.^34^ We evaluated and visualized thermodynamic potentials and sulfur models using our in-house code ThermoPot.^25^ This analysis is available in an online repository.^26^ ThermoPot is a general-purpose and material-agnostic code which postprocesses first-principles data. We identified low-energy materials in the Ba–Zr–S system using the total energies reported on the Materials Project.^38^ More details on the database search are provided in the Supporting Information.

### Quantum Chemical Calculations

First-principles calculations were carried out with the all-electron numerical atom-centered orbital code FHI-aims. All calculations were performed using the default “tight” basis set in FHI-aims, which extends the minimal set of occupied orbitals with six additional functions.^39^ For reciprocal space sampling, a Monkhorst–Pack grid was used with a minimum *k*-spacing of 0.2 Å^–1^. For the self-consistent field cycles the charge density was converged to an accuracy of 10^-7^ and forces to 10^–6^ eV Å^–1^.

Equilibrium crystal structures were obtained using a parametrically constrained geometry relaxation implemented in the AFLOW Library interface to FHI-aims.^40^ Geometry relaxations for solid materials were performed using the generalized gradient approximation (GGA) PBEsol^36^ functional. The structures were relaxed until the maximum force component was below 5 × 10^–3^ eV Å^–1^. We do not include a correction for van der Waals interactions, but note that our calculated value for interlayer spacing in ZrS_2_ (3.63 Å) is within 0.5% of the experimentally reported value at room temperature (3.65 Å, ICSD entry 76037). The initial geometries for the sulfur gas species (S_2_ and S_8_) were obtained from the NIST Computational Chemistry Comparison and Benchmark Database.^33^

Harmonic phonon dispersions were evaluated using the finite-difference method, as implemented in Phonopy,^41^ with a 0.01 Å step-size. The forces were evaluated using the PBEsol functional. Harmonic phonon theory for the calculation of free energies fails when there are phonon modes with imaginary frequency, as the contribution to the vibrational partition function is ill defined. In this case, anharmonic contributions need to be considered. In this study all materials which we predict to be thermodynamically stable are also kinetically stable (i.e., no phonon modes with imaginary frequency). The Supporting Information contains phonon bandstructures for the binary materials included in this study (Figures S37–S45).

Predictions for phase stability are highly sensitive to the calculated total energies from DFT. To allow comparison between layered and three-dimensional Ba–Zr–S phases with diverse bonding, we evaluated the total electronic energies using the meta-GGA SCAN functional.^35^ A comparison against results generated using the hybrid HSE06^37^ and GGA PBEsol^36^ functionals are provided in the Supporting Information. All other inputs were set to the default value within FHI-aims. The Supporting Information contains electronic bandstructures (Figures S30–S36) for BaZrS_3_ and the binary materials included in this study.

## Results

### BaZrS_3_ Formation from Solid Materials

First, we consider the formation of perovskite from its constituent elements in standard conditions, *T* = 298 K and *P* = 1 × 10^5^ Pa. At this temperature all precursors take solid form, including sulfur in the α-phase:
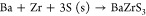
R1

**Figure 2:**
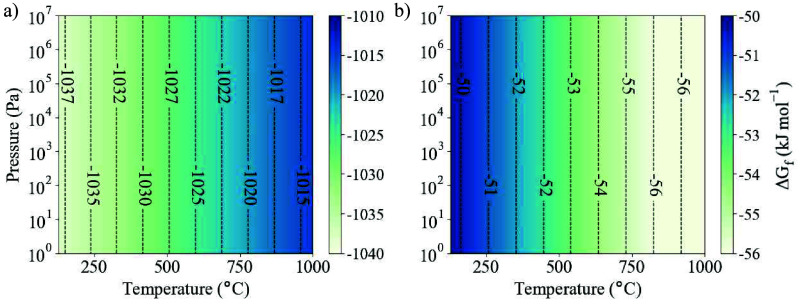
Gibbs free energy of formation of BaZrS_3_ from (a) its constituent elements (Reaction R1) and (b) binary precursors in their formal oxidation states (Reaction R2). As there is no sulfur gas component, the pressure results from an inert gas or mechanical force.

We predict a formation enthalpy of −2.18 eV/atom. We show that Reaction R1 is thermodynamically feasible over a range of temperatures and pressures in Figure 2a. Due to the absence of a gas phase and assumed incompressibility of each solid, the Gibbs free energy of perovskite formation, Δ*G*_f_, is weakly dependent on pressure. We conclude that across a wide range of synthesis conditions, BaZrS_3_ is stable with respect to its elemental components.

To consider more experimentally relevant precursors, we model Ba and Zr in their formal oxidation states of +2 and +4, respectively:
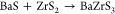
R2Our results show that there is a thermodynamic driving force toward the formation of BaZrS_3_ across the full temperature and pressure range (Figure 2b). The binary precursors are more stable than the elemental species, leading to a reduction in the net energy released. As the temperature increases, BaZrS_3_ becomes increasingly stable against degradation into the binary phases. This follows from a relatively flat phonon dispersion for BaZrS_3_ at frequencies beneath 2.5 THz, leading to an increased density of states and vibrational entropy (Figures S5–S7).^42^

### Reaction Thermodynamics in Sulfur Gas

We have demonstrated that it is thermodynamically favorable to form BaZrS_3_ from BaS and ZrS_2_, but the literature shows that there are severe kinetic limitations which limit this process at moderate temperatures (∼600 °C). Increasing the synthesis temperature overcomes the kinetic barrier, with crystalline material typically grown at 800 °C and above.^10,16^ Sulfur partial pressure is an additional parameter which has been tuned to accelerate the reaction dynamics of BaZrS_3_. In particular, overstoichiometric amounts of sulfur are shown to promote the formation of perovskite at growth temperatures suitable for thin-film synthesis (<600 °C).^17−19,43−45^

A common synthesis approach is to start with sulfur in the solid state (α-sulfur), followed by heat treatment in a sealed ampule. During this annealing step, the sulfur sublimates to form sulfur gas. Assuming the gas is in equilibrium, the ideal gas law can be applied to determine the sulfur partial pressure from the annealing temperature, ampule volume, and sulfur amount. Another common assumption made in this calculation is that the sulfur gas is composed of a single allotrope (no allotrope mixing). However, in actuality gaseous sulfur forms a range of open and closed S_*n*_ species with equilibrium allotrope ratios that are highly sensitive to temperature and pressure.^22,23^ Furthermore, annealing may occur in nonequilibrium conditions resulting from the experimental setup, such as gas flow, gas escape, or temperature gradients.^46^ Experimental insight into reaction conditions required for BaZrS_3_ synthesis is limited by the challenges associated with in situ monitoring of sulfur gas partial pressure during material synthesis.

For a system in equilibrium, quantum chemical modeling is deemed to give the most accurate understanding of sulfur gas constitution.^47^ Jackson et al. used a global structure search and first-principles lattice dynamics to identify the equilibrium ratios of 13 low-energy sulfur gas allotropes.^23^ They concluded that the dominant components are S_2_ and S_8_. The trend is for high-*n* (*n* = 8) species to dominate at low temperatures and high pressures, and low-*n* (*n* = 2) species to dominate at high temperatures and low pressures. This is in agreement with experimental measurements.^22,23,47^ The transition temperature at which the dominant phase changes (corresponding to the point at which *μ*_S_2__ = *μ*_S_8__) is highly pressure-dependent, with higher pressures corresponding to higher transition temperatures. In addition, higher pressures correspond to a wider temperature range in which contributions from the cyclic allotropes S_4_–S_7_ are significant.^23^

In this work, we consider three sulfur gas compositions: (i) single-component S_2_ (the smallest allotrope); (ii) single-component S_8_ (the largest allotrope); and (iii) an equilibrium mixture of the S_2_–S_8_ allotropes (13 species in total),^23^ denoted S_mix_. The enthalpy change when converting between sulfur allotropes is largest for S_2_ and S_8_ at moderate temperatures,^48^ so that these systems reflect the extremes of behavior that might be expected for (out of equilibrium) single-allotrope systems. S_mix_ corresponds to a gas that is in equilibrium, which we assume here provides the most accurate model for comparison against the experiment.

**Figure 3:**
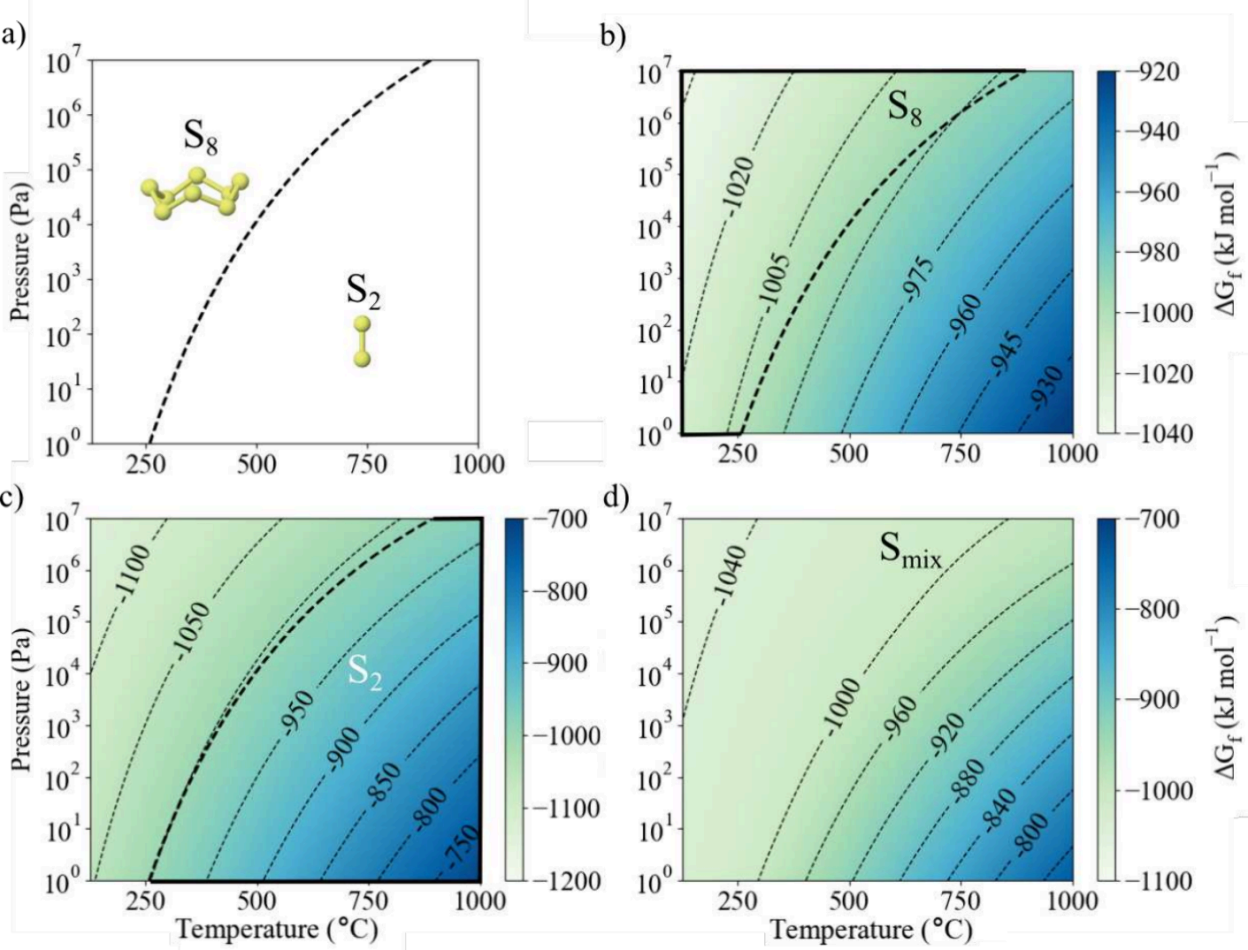
(a) The coexistence curve for single allotrope S_2_ vapor and single allotrope S_8_ vapor. The dashed line indicates the coexistence curve where the chemical potential *μ* of the sulfur gas allotropes on a per-atom basis are equal, *μ*_S_8__ = *μ*_S_2__. At lower temperatures and higher pressures, S_8_ dominates, and at higher temperatures and lower pressures, S_2_ dominates. The data reproduces that already published by Jackson et al.^23^ (b) Gibbs free energy of BaZrS_3_ formation from solid Ba, Zr, and S_8_ (Reaction R3 in the main text). The dashed and heavy solid lines indicate the region where the S_8_ allotrope dominates. (c) Gibbs free energy of BaZrS_3_ formation from solid Ba, Zr, and S_2_ (Reaction R4). The dashed and heavy solid lines indicate the region where the S_2_ allotrope dominates. (d) Gibbs free energy of BaZrS_3_ formation from solid Ba, Zr, and S_mix_ (Reaction R5). The chemical potential of S_mix_ incorporates the effects of S_2_ to S_8_ allotrope mixing.^23^

To demonstrate our approach, we consider perovskite formation from elemental compounds with sulfur in the gas phase. We consider an atmosphere with sulfur vapor only, so that the sulfur partial pressure is equal to the total pressure. For the higher-pressure, lower-temperature regime, the S_8_ allotrope is more stable than the S_2_ allotrope, with *μ*_S_8__ < *μ*_S_2__, where *μ* is the chemical potential of a single atom (Figure 3a). As such, in this regime we expect perovskite formation in equilibrium with the S_8_ allotrope to be the more relevant reaction (Figure 3b):
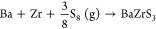
R3

For the lower-pressure, higher-temperature regime where *μ*_S_2__ < *μ*_S_8__ the equilibrium with the S_2_ allotrope is more relevant (Figure 3c):
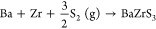
R4

The range of experimental conditions used for BaZrS_3_ (precursor) synthesis is coincident with the coexistence curve in Figure 3a, so we expect allotrope mixing to impact reaction thermodynamics. Furthermore, we require a model that is valid across the temperature and pressure ranges used for synthesis. As such, we consider equilibrium with S_mix_ to be the most relevant reaction for comparison against experimental results:
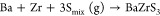
R5

For all sulfur gas compositions, we find that the formation of BaZrS_3_ is thermodynamically favored. As expected, increased sulfur partial pressure stabilizes the perovskite, as the gas species favors entry into a low-pressure environment. The temperature dependence is also more acute compared to the formation from α-sulfur. This follows from the free translational and rotational motion of a gas molecule leading to higher entropy.

We find that the sulfur allotrope can have a noticeable impact on Δ*G*_f_. For example, under typical processing conditions (*T* = 500 °C, *P* = 1 × 10^2^ Pa), the predicted Δ*G*_f_ is 34 kJ mol^–1^ less when forming perovskite in equilibrium with S_8_ (Reaction R3) compared to an equilibrium with S_2_ (Reaction R4). This is consistent with S_2_ being the stable allotrope at *T* = 500 °C, *P* = 1 × 10^2^ Pa.

To understand the impact of allotrope mixing, we compare the single-species models (Figures 3b and 3c) against the mixed-allotrope model (Figure 3d). As expected, in the lower-temperature and higher-pressure regions where the S_8_ allotrope dominates, there is a close agreement between Δ*G*_f_ calculated for a reaction with the single allotrope S_8_ and the mixed allotrope S_mix_. In the higher-temperature and lower-pressure region where the S_2_ allotrope dominates, there is closer agreement between S_2_ and S_mix_ than S_8_ and S_mix_. The mixed- and single-allotrope predictions deviate most strongly in the region where the sulfur model predicts significant mixing between allotropes, which encompasses the region typically targeted for BaZrS_3_ formation (Figures S8 and S9). This indicates that the extent of allotrope mixing may have an impact on the thermodynamic feasibility of reaction processes.

### Formation of Sulfur-Rich Binary Precursors

The critical temperature for accelerated perovskite growth coincides with the low melting point of the intermediate phase BaS_3_ at 554 °C.^20^ Following this, it is proposed that BaS_3_ acts as a liquid flux which overcomes the kinetic barriers associated with solid-state precursors.^1,17−19^ An additional source of sulfur during annealing gives access to these sulfur-rich binary phases. Although experimental studies have started to explore the conditions under which BaS_3_ is formed,^17−19^ the upper and lower limits of sulfur partial pressure is currently undefined. ZrS_3_ formation has also been reported during BaZrS_3_ synthesis, with several studies suggesting that forming ZrS_3_ as a reaction intermediate can hinder the formation of perovskite.^19,43^

To predict which solid binary materials are most stable at a particular temperature and sulfur partial pressure, we assume that precursors are in the (Ba,Zr)-S system and that the reaction occurs in equilibrium with a mixed-allotrope sulfur atmosphere. For example, to compare the stability of BaS and BaS_2_, we calculate Δ*G*_f_(*T*, *P*) for the following reaction:
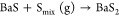
R6If Δ*G*_f_ for Reaction R6 is negative, we predict that BaS_2_ will form in preference to BaS at that particular temperature and pressure. We conduct this analysis for all materials reported to be within 0.5 eV of the convex hull when calculated using ground-state DFT (see the Supporting Information for more details on the database search). For Ba–S, this corresponds to calculating the relative stability of BaS, Ba_2_S_3_, BaS_2_, and BaS_3_. For Zr–S, we consider ZrS, Zr_3_S_4_, ZrS_2_, and ZrS_3_.

**Figure 4:**
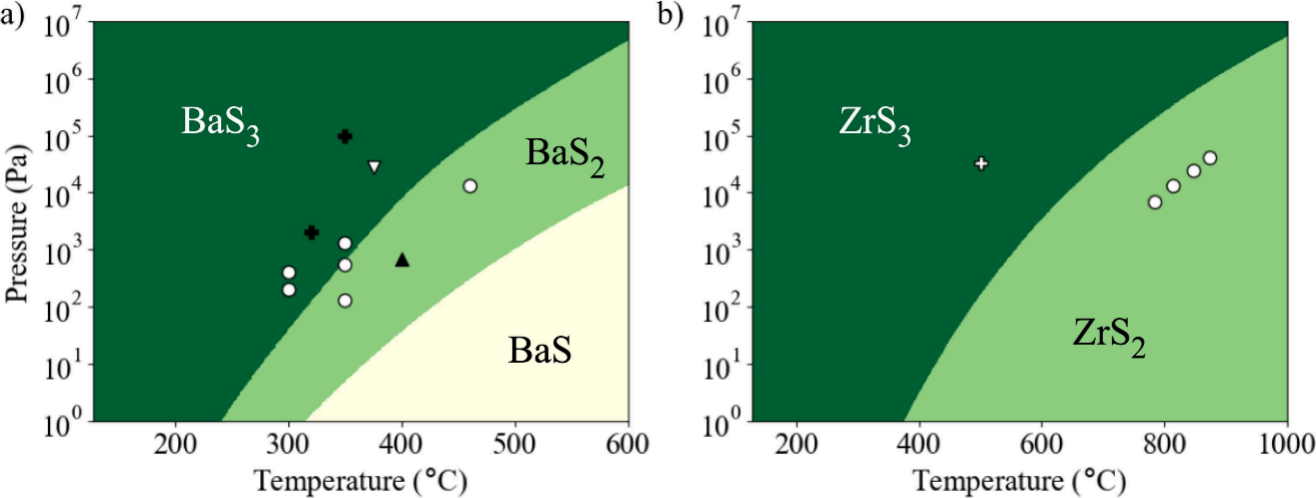
Diagram displaying the most stable compounds when in equilibrium with sulfur vapor. (a) Ba–S materials. The scatter points are taken from published experimental data. White-filled shapes correspond to BaS_3_ formation: circles are from ref (17) and triangles are from ref (18). The black-filled triangle corresponds to BaS_2_ and the black-filled crosses correspond to BaS, both from ref (17). Note that we do not consider the liquid phases of BaS_*x*_, which are known to form above 554 °C.^20^ (b) Zr–S materials. The scatter points are taken from published experimental data, with the cross from ref (18) denoting ZrS_3_ formation. The circles from ref (49) denote the points at which ZrS_3_ decomposes to ZrS_2_.

The most stable materials are displayed as a function of temperature and pressure in Figure 4. For the Ba–S system, we find that the sulfur partial pressure has a marked impact on stability at moderate temperatures. For example, at 400 °C BaS_2_ formation is favorable between 4 × 10^1^ to 1 × 10^4^ Pa, with BaS formed below this range and BaS_3_ formed above. As the temperature increases, the partial pressures required for sulfur-rich compounds to form also increase. Ba_2_S_3_ is predicted to be unstable across the temperature and pressure ranges considered in this analysis.

There is limited data available for BaS_3_ formation in an evacuated ampule with a sulfur source. Yang et al. annealed BaS at 400 °C and found conversion into BaS_3_.^19^ Vincent et al. used a similar approach and found conversion to BaS_3_ at 375 °C and at an estimated sulfur partial pressure of 0.28 × 10^5^ Pa.^18^ This measurement is within the range of pressures we predict for BaS_3_ stability. After removing the sulfur source from the ampule, Vincent et al. observed decomposition into BaS_2_ at 575 °C. This indicates that BaS_3_ is not stable at moderate temperatures and reduced partial pressure, in line with our predictions.

Freund et al. annealed BaS in a sulfur–nitrogen atmosphere across a range of temperatures (250 to 460 °C) and total pressures (1.3 × 10^2^ to 1 × 10^5^ Pa).^17^ They report BaS_3_ formation at pressures within and below our predicated range. They also found that for a given temperature, if the pressure was too high, then no reaction occurred and BaS remained. This is contrary to equilibrium gas behavior, suggesting a change in reaction conditions or the onset of a competing reaction, which is not captured in our model. Comparotto et al. report the synthesis of BaZrS_3_ across a range of sulfur partial pressures.^10^ They report improved crystallinity at higher sulfur partial pressures and propose that this may be a consequence of BaS_3_ liquid-flux formation. However, the upper bound for pressure is 5 Pa at 590 °C, which is significantly below the lower bound we predict for BaS_3_ formation. We note that as we do not consider BaS_3_ in the liquid phase, which might be stabilized at this temperature for lower partial pressures.

For the Zr–S system in Figure 4b, our model predicts that ZrS_2_ and ZrS_3_ are the only thermodynamically stable materials. Experimental data for the decomposition of ZrS_3_ into ZrS_2_ and sulfur gas lies within 1 order of magnitude of our model (Figure 4b).^49^ The scaling relationship between decomposition temperature and pressure is remarkably close to what we predict, with higher temperatures corresponding to higher pressures. Vincent et al. report conversion of a ZrH_2_ precursor to ZrS_3_ at 500 °C and a sulfur partial pressure of 3.3 × 10^4^ Pa, which lies in the predicted region of ZrS_3_ formation.^18^

We find that ZrS_3_ is formed across a wider range of temperatures and pressures than BaS_3_. For example, at 500 °C, ZrS_3_ is formed above 2 × 10^2^ Pa, compared to 3 × 10^5^ Pa for BaS_3_. The consequence of this is that in certain conditions BaS_2_ and ZrS_3_ will coexist. Yang et al.^19^ report that after combining BaS_3_ and ZrS_2_ powders (with no sulfur excess) in a vacuum-sealed ampule, a reaction is initiated to form BaS_2_ and ZrS_3_ at 500 °C. This is in agreement with our prediction that Δ*G*_f_ is −15.8 kJ mol^–1^ for the corresponding reaction:
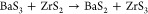
R7with sulfur vapor proposed as a reaction intermediate (see Reactions R1 and R2 in the Supporting Information).

We find that the positions of the coexistence curve(s) are sensitive to sulfur vapor composition, with S_8_ hindering the formation of BaS_3_ across the temperature and pressure ranges used for synthesis (Figures S11 and S12). This suggests that sulfur allotropes may be a significant factor in the thermodynamics of BaS_3_ formation; equilibrium and nonequilibrium sulfur vapors may lead to different chemical behaviors.

In conclusion, we find that there is high sensitivity to the sulfur partial pressure, and this must be carefully controlled to access the desired binary intermediates and avoid the undesired. At 500 °C, a sulfur partial pressure above 3 × 10^5^ Pa is required to form BaS_3_. At this point, it also becomes thermodynamically favorable to form ZrS_3_, which is reported to hinder BaZrS_3_ formation. As such, our model supports the recently reported strategy of using a liquid BaS_3_ precursor rather than an annealing step with an additional sulfur source.^19^

### BaZrS_3_ Stability against Degradation into Sulfur-Rich Binaries

Several experimental studies have established that if the partial pressure of sulfur is too high during annealing, ZrS_3_ growth is favored at the expense of BaZrS_3_ formation. Two mechanisms have been proposed for this.^1^ High partial pressures may cause BaZrS_3_ to become unstable:
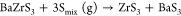
R8
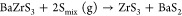
R9Or, alternatively, ZrS_2_ forms ZrS_3_ in a sulfur-rich atmosphere:
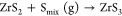
R10with ZrS_3_ mass transport limiting perovskite formation.

**Figure 5:**
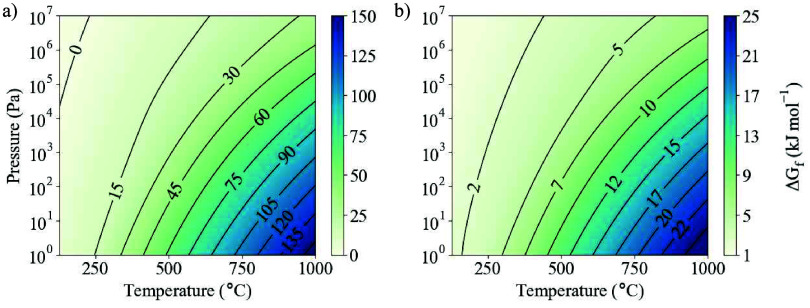
Gibbs free energy of BaZrS_3_ degradation when in equilibrium with a mixed-allotrope sulfur gas. (a) Degradation into ZrS_3_ and BaS_3_ (Reaction R8). (b) Degradation into ZrS_3_ and BaS_2_ (Reaction R9).

We have demonstrated in the previous section that ZrS_3_ can form across a wide range of temperatures in a sulfur-rich atmosphere. In Figure 5, we consider Reactions R8 and R9. We find that BaZrS_3_ in equilibrium with sulfur gas is stable against degradation into ZrS_3_. This result is supported by a recent study demonstrating a complete conversion of BaS_3_ and ZrS_3_ into BaZrS_3_ for a reaction time of 3 days.^19^ We note that while BaZrS_3_ might be stable with respect to binary materials in a sulfur-rich environment, the increased chemical potential of sulfur may lead to other unwanted effects; for example, the formation of sulfur interstitial defects. Of particular concern is the S_Zr_ antisite substitution, which is predicted to have a low formation energy and electronic levels within the BaZrS_3_ band gap.^13^

In the Supporting Information, we consider perovskite degradation into all binary and elemental precursors within 0.5 eV of the convex hull. In all cases, BaZrS_3_ remains stable. When BaZrS_3_ is formed in an atmosphere with unstable single-allotrope vapor S_2_ (Figure S15), we predict degradation only at pressures above the saturation vapor pressure for sulfur, where our ideal gas model is no longer valid.^50^

## Conclusions

In this work, we have considered reported phases in the Ba–Zr–S system within 0.5 eV of the convex hull. Our results demonstrate the thermodynamic feasibility of forming perovskite at moderate temperatures; we predict that up to a temperature of 1000 °C, BaZrS_3_ is stable against decomposition into solid binary or elemental competing phases. Importantly, this is true when annealed in mixed-allotrope (equilibrium) sulfur vapor at high partial pressures.

Our model predicts that several sulfr-rich BaS_*x*_ and ZrS_*x*_ materials can be formed when the binary systems are annealed in sulfur vapor. A general trend is that to form a more sulfur-rich species, the temperature must be reduced or the sulfur partial pressure increased. At 500 °C, a sulfur partial pressure above 3 × 10^5^ Pa is required to form BaS_3_. At any temperature and pressure where BaS_3_ is formed, ZrS_3_ is also predicted to form. For this reason, our results support the idea of using BaS_3_ and ZrS_2_ precursors for fast perovskite synthesis, before kinetically limiting ZrS_3_ can be formed.

We have focused our analysis on the most commonly reported competing phases or reaction intermediates and emphasize that thermodynamic free energies for all possible product and reactant combinations reported in this study can be predicted using our computational notebook^26^ and ThermoPot software.^25^ ThermoPot is material-agnostic, so it can be used to postprocess first-principles calculations for other systems; the same approach outlined here could be applied to alloy chalcogenide perovskites, for example. Extension to other gas species is possible where there is published thermochemical data.

To widen the material space for this system or incorporate the effects of disorder, recent advances in computational modeling could be applied. For example, thermodynamic integration techniques to incorporate liquid BaS_*x*_ phases or statistical methods to quantify the configurational entropy term associated with Ruddlesden–Popper phase intermixing. Oxide materials might also play a role in BaZrS_3_ formation, with ZrO_2_ formation potentially acting as a Zr “sink”.

Compared to experimental measurements, our model overestimates the partial pressure at which BaS_3_ and ZrS_3_ begins to form. This motivates further studies to close the gap between theory and experiment. On the theory side, the ab initio model for lattice dynamics could be generalized to include the effects of thermal expansion (using the quasi-harmonic approximation) or higher-order lattice anharmonicity (using a temperature-dependent effective potential, for example). For experiments, improved experimental measurements and control of temperature and sulfur partial pressure, with analysis that accounts for allotrope mixing, would provide more accurate measurements for comparison.

A key result is that the constitution of sulfur vapor has a significant impact on reaction thermodynamics. It is difficult to maintain a constant temperature and sulfur partial pressure during chalcogenide material synthesis, and as has been seen for CZTS,^51^ the proportion of sulfur allotropes may fluctuate depending on sample position, dwell time, and the particulars of the experimental setup.^46^ As a result, there could be challenges for experimental reproducibility, with fluctuations in the rate and feasibility of perovskite formation for comparable setups. This insight can be transferred to other sulfide materials with phase equilibria in temperature and pressure regions with significant allotrope mixing and motivates further work in the characterization and control of the sulfur vapor.

## Data Availability

Calculation input and output files (including the relaxed geometries, total energies, and phonon data) are available in both project-specific^26^ and NoMaD^27^ repositories. Computational notebooks to reproduce the results reported here are also available in the project-specific repository.

## Supporting Information Available

Phonon dispersions, electronic band structures, and additional computational details and analysis (PDF)
