# Supplementary material for: A First-Principles Thermodynamic Model for the Ba–Zr–S System in Equilibrium with Sulfur Vapor
Source: ACS Appl Energy Mater. 2024 Apr 5;7(24):11326–33. doi: 10.1021/acsaem.3c03208 (PMC11672234; doi:10.1021/acsaem.3c03208)
Supplement: Supplementary file 1 — ae3c03208_si_001.pdf [file ae3c03208_si_001.pdf]

Supporting Information for 'A first-principles thermodynamic model for the Ba–Zr–S system in equilibrium with sulfur vapour'

Prakriti Kayastha, Giulia Longo, and Lucy D. Whalley*

Department of Mathematics, Physics and Electrical Engineering, Northumbria University, Newcastle upon Tyne, NE1 8QH, United Kingdom

E-mail: l.whalley@northumbria.ac.uk

Database search

From the Materials Project v2022.10.28,[1] we query for the binary Ba-S and Zr-S systems and the ternary Ba-Z-S systems. We select the compounds that i) have been experimentally reported on the ICSD;[2] ii) lie within 0.5 eV of the convex hull (as calculated using the default GGA functional); iii) have primitive cells containing less than 15 atoms.

Table S1: Materials selected through our database search

Formula	Space Group	Materials Project ID	ICSD no.
BaS	$Fm\bar{3}m$	mp-1500	2004
BaS_2	$C2/c$	mp-684	42134
BaS_3	$P\bar{4}2_1m$	mp-239	23637
BaS_3	$P2_12_12$	mp-556296	26765
Ba_2S_3	$I4_1md$	mp-28978	70058
ZrS	$Fm\bar{3}m$	mp-1925	52224
ZrS	$P4/nmm$	mp-7859	24754
ZrS_2	$P\bar{3}m1$	mp-1186	76037
ZrS_3	$P2_1/m$	mp-9921	42073
Zr_3S_4	$Fd\bar{3}m$	mp-1103820	108734
$BaZrS_3$	$Pnma$	mp-540771	23288
Ba_2ZrS_4	$I4/mmm$	mp-3813	69853
$Ba_3Zr_2S_7$	$P4_2/mnm$	mp-8570	264213
$Ba_3Zr_2S_7$	$I4/mmm$	mp-9179	75241
$Ba_4Zr_3S_{10}$	$I4/mmm$	mp-14883	72656

We have already established that $BaZrS_3$ decomposes to the Ruddlesden-Popper (RP) phases and ZrS_2 at high temperatures only (>1200 K).[3] In addition, an equilibrium with sulfur vapour will make the formation of relatively sulfur-poor RP phases less thermodynamically favourable. In this study we consider perovskite formation at moderate temperatures in sulfur vapour, so we do not include an analysis of RP-phase formation.

The total energies used to construct the convex hull correspond to the total electronic energies calculated using ground-state Density Functional Theory. A 0.5 eV cutoff above the convex hull was chosen as this is reported to include the 90th percentile of all metastable materials reported within the Materials Project.[4,5] To assess if this is likely to capture all of the materials which might be stabilised within the temperature and pressure ranges we are

considering, we evaluated the PV and TS terms for each binary material in Table S1 using first-principles thermodynamics, as outlined in the methods section of the main text. We used the resulting Gibbs free energies and pymatgen[6] to plot the convex hull at 1000 °C. For incompressible solids the effect of pressure is negligible; in this case we consider $P = 1\,\text{Pa}$. In Figures S1 to S4 we show that for both systems the largest change in energy relative to the convex hull is less than 0.5 eV, which suggests our criteria for the selection of ground-state materials is reasonable. All energies are calculated using the SCAN exchange-correlation functional.[7]

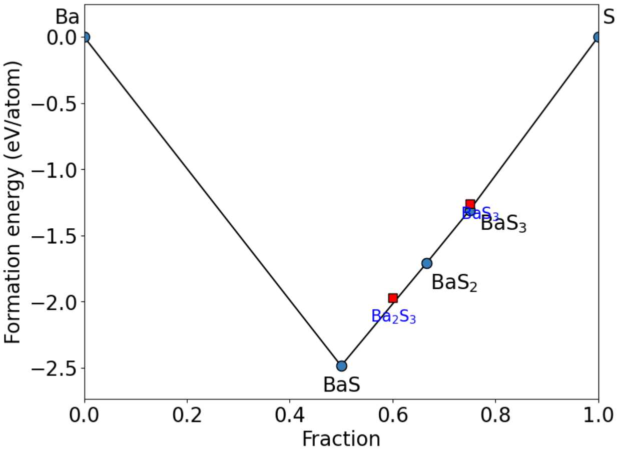

Figure S1: Convex hull at 0 K for materials in the Ba-S system. Red points denote materials which are metastable (above the convex hull).

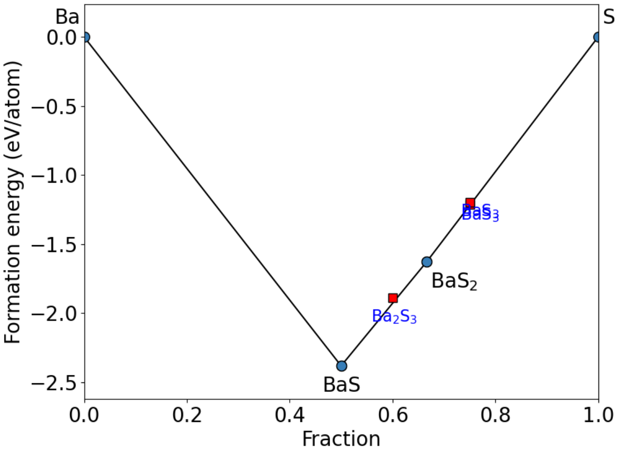

Figure S2: Convex hull at 1273 K for materials in the Ba-S system. Red points denote materials which are metastable (above the convex hull).

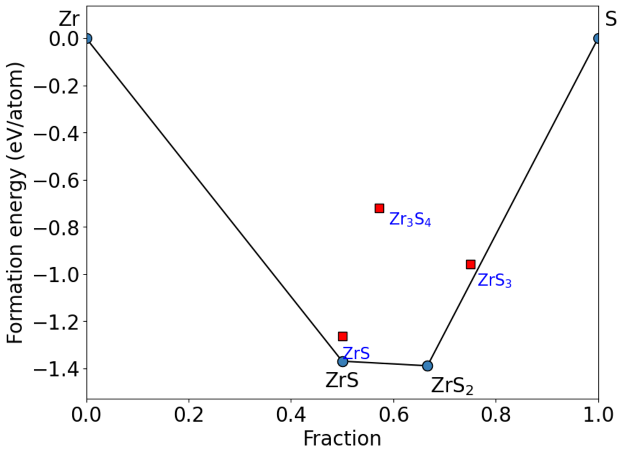

Figure S3: Convex hull at 0 K for materials in the Zr-S system. Red points denote materials which are metastable (above the convex hull).

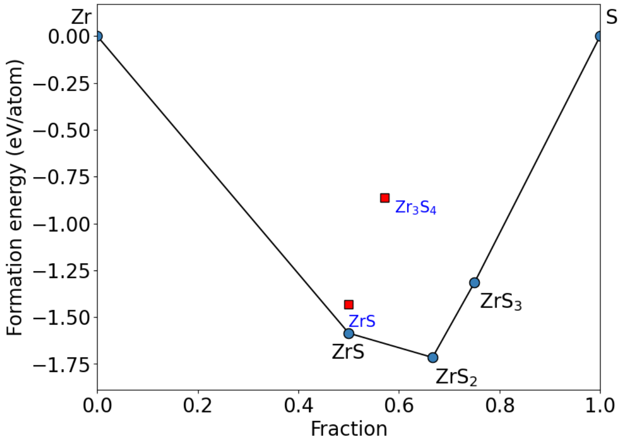

Figure S4: Convex hull at 1273 K for materials in the Zr-S system. Red points denote materials which are metastable (above the convex hull).

Entropic contributions to the Gibbs Free Energy of formation

In Fig 2b of the main text we see that the perovskite product becomes increasingly stabilised with temperature for the reaction $BaS + ZrS_2 \rightarrow BaZrS_3$. This stems from a net entropy gain during perovskite formation. This can be monitored by extracting the S term from our lattice dynamics calculations; see Figure S5 for the entropy of each component, and Figure S7 for the change in entropy during perovskite formation. We postulate that the relatively large perovskite entropy term results from the high density of flat bands beneath 2.5 THz in Figure S45, which likely leads to an increased density of states at lower phonon frequencies. In Fig 2a of the main text we see the opposite behaviour: perovskite formation from elemental compounds (Figures S6 and S7) becomes less favourable with increasing temperature (a net entropy loss).

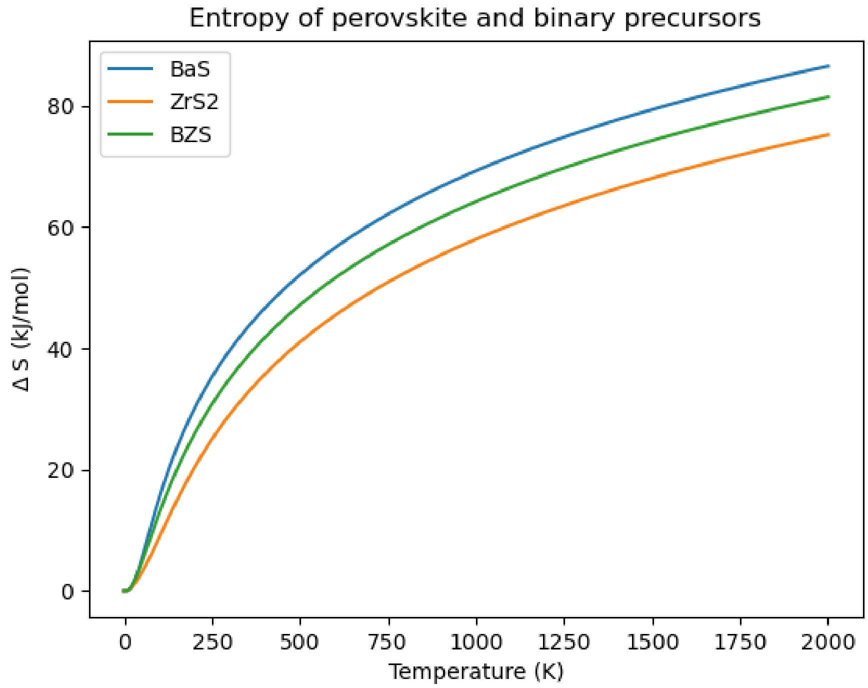

Figure S5: Entropy of $BaZrS_3$ perovskite and binary precursors calculated using first principles lattice dynamics. BZS denotes $BaZrS_3$.

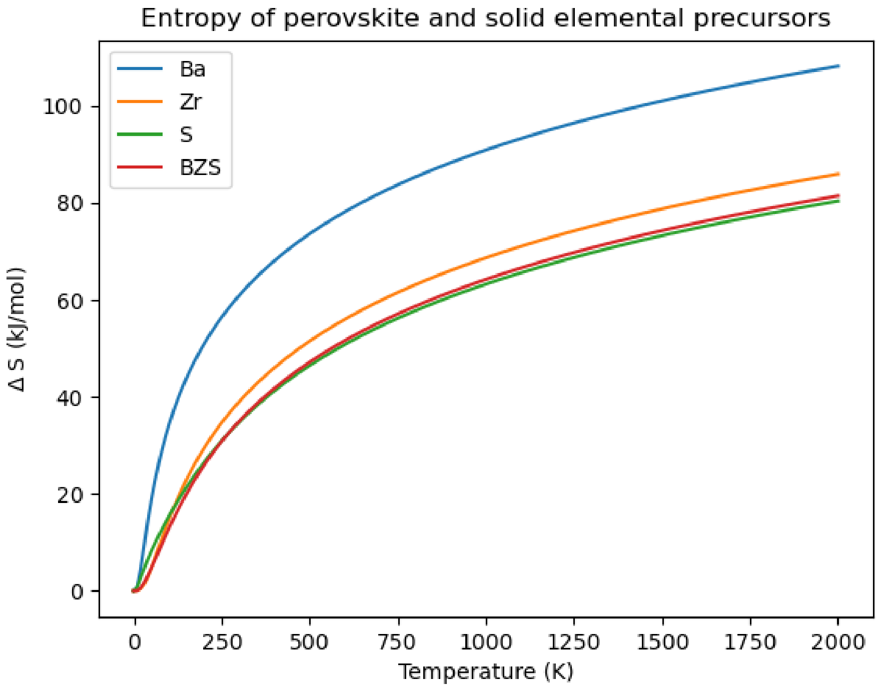

Figure S6: Entropy of $BaZrS_3$ perovskite and solid elemental precursors calculated using first principles lattice dynamics. BZS denotes $BaZrS_3$.

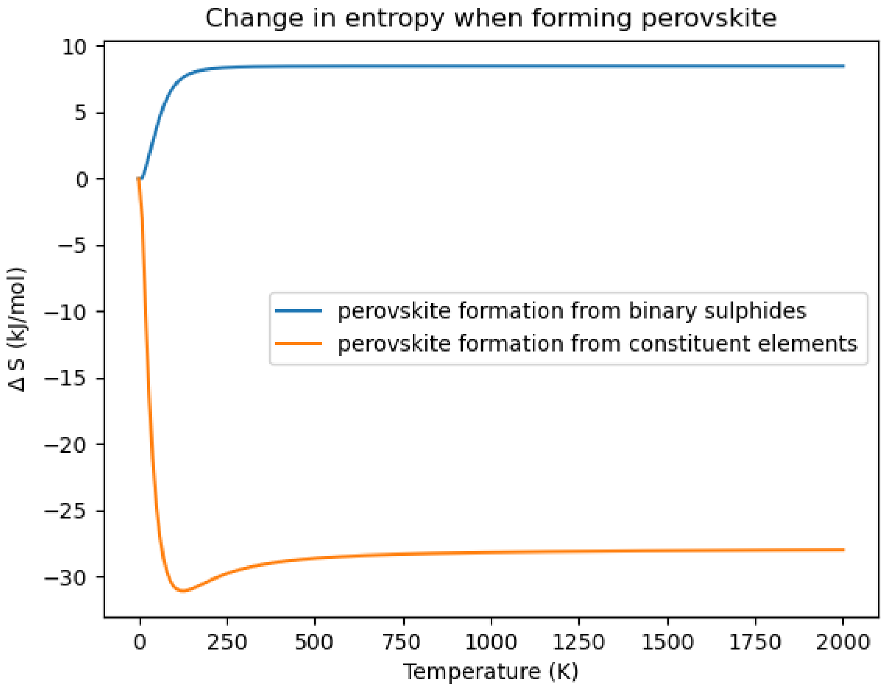

Figure S7: Change in entropy when forming $BaZrS_3$ perovskite from solid elemental precursors or binary precursors. BZS denotes $BaZrS_3$.

Comparison between the single-allotrope and mixed-allotrope models

In Figure S8 we compare the predicted Gibbs free energies of a single allotrope S_2 vapour with a mixed-allotrope vapour. In Figure S9 we compare the predicted Gibbs free energies of a single allotrope S_8 vapour with a mixed-allotrope vapour. As outlined in the methods section of the main text the Gibbs free energy of the single-allotrope vapour is calculated using first-principles methods and tabulated experimental data, whilst the Gibbs free energy of the mixed-allotrope vapour is calculated using a previously published sulfur model[8] parameterised with the hybrid PBE0 functional.[9]

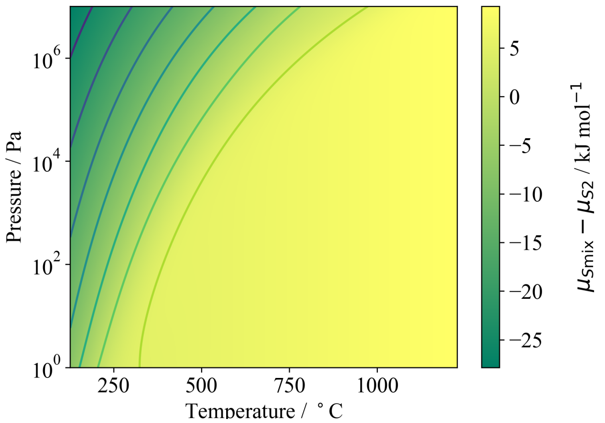

Figure S8: Comparison of the Gibbs free energy associated with a single allotrope sulfur vapour S_2 and mixed allotrope S_{mix}. The pressure is the partial pressure of the sulfur gas.

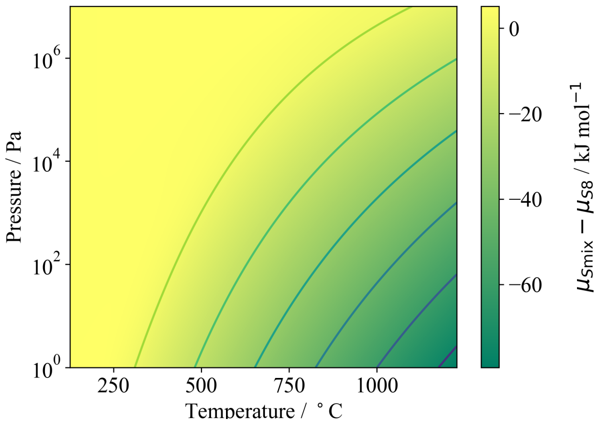

Figure S9: Comparison of the Gibbs free energy associated with a single allotrope sulfur vapour S_8 and mixed allotrope S_{mix}. The pressure is the partial pressure of the sulfur gas.

As would be expected, in both Figure S8 and Figure S9 the models begin to diverge in the region where the published sulfur model predicts significant mixing of allotropes S_2 to S_8.[8] This coincides with the experimental conditions typically reported for $BaZrS_3$ synthesis via annealing in sulfur vapour (temperatures between 500 °C to 600 °C, and pressures between 1×10^3 Pa to 1×10^5 Pa). This highlights the importance of moving beyond the approximation of a single sulfur species in models for modelling perovskite formation. The sulfur model is referenced to an S_8 species so that there is closer agreement between S_8 and S_{mix} (in the low-T, high-P S_8-rich regions) than S_2 and S_{mix} (in the high-T, low-P S_2-rich regions). Where the respective allotrope does not dominate, S_{mix} is the more stable system (with a negative chemical potential of larger magnitude).

Sulfur transfer between binary precursors

Yang et al. report that after combining BaS_3 and ZrS_2 powders (with no sulfur excess) in a vacuum-sealed ampule a reaction is initiated to form BaS_2 and ZrS_3 at 500 °C.[10] In Figure S10 we present the change in Gibbs free energy for the reaction $BaS_3 + ZrS_2 \longrightarrow BaS_2 + ZrS_3$. Across the whole temperature and pressure range the formation of BaS_2 and ZrS_3 is predicted to be thermodynamically favourable.

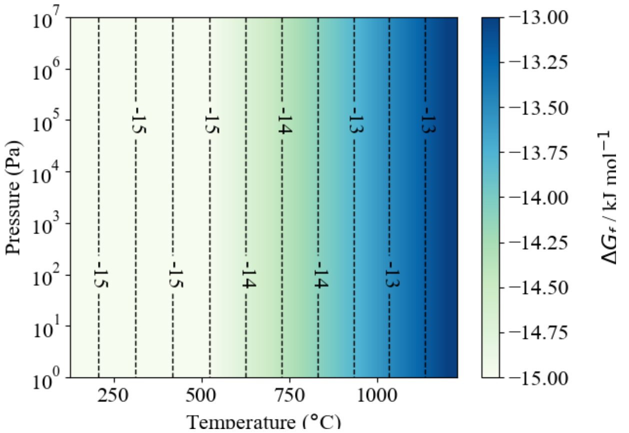

Figure S10: Gibbs free energy of formation for the reaction $BaS_3 + ZrS_2 \longrightarrow BaS_2 + ZrS_3$. As there is no sulfur-gas component, the pressure results from an inert gas or mechanical force.

It is possible that sulfur gas is formed as an intermediate, so that the reaction is a two-step process:

$$BaS_3 + ZrS_2 \longrightarrow BaS_2 + S(g) + ZrS_2 \quad \text{(R1)}$$

$$BaS_2 + S(g) + ZrS_2 \longrightarrow BaS_2 + ZrS_3 \quad \text{(R2)}$$

In Figure 4a of the main text we show that R1 is a forward reaction below a sulfur partial pressure of 1 bar at 500 °C, which is realistic when there is no additional sulfur source. Figure 4b shows that R2 is stabilised at sulfur partial pressures above 1 mbar. Following formation of ZrS_2 and BaS_3, there will be a kinetically limited reaction to form the final product $BaZrS_3$, as outlined in the main text.

We conclude that to stabilise the binary precursors BaS_3 and ZrS_2 at 500 °C a sulfur partial pressure above 1 bar should be maintained so as to prevent R1.

Relative stability of binary precursors in equilibrium with S_2 or S_8 vapours

In the main text we present results for the binary Ba-S and Zr-S systems in equilibrium with a mixed-allotrope sulfur vapour. Here we compare the results for each system in equilibrium with either single-allotrope S_2 or single-allotrope S_8 vapours. A direct comparison between the mixed- and single-allotrope results is difficult as each simulation uses a different methodology. However we can compare single-allotrope S_2 against single-allotrope S_8 to better understand the range of behaviour that might be expected for systems that are out of thermodynamic equilibrium.

We find that the position of the co-existance curves (where two systems have equal Gibbs free energy) are sensitive to sulfur vapour type, with S_2 vapour promoting the formation of BaS_3 across an increased temperature and pressure range compared to S_8 (Figures S11 and S12). This suggests that a high-temperature sulfur source producing S_2 may promote the formation of sulfur-rich species if it can react with the metal precursor before equilibrating to a more stable, mixed-allotrope vapour. For the Zr-S system we find that S_2 vapour hinders the formation of sulfur rich ZrS_3. For example, ZrS_3 is thermodynamically favourable across all partial pressures at 500 °C in S_8, whilst this is reduced to 350 °C in S_2 (Figures S13 S14).

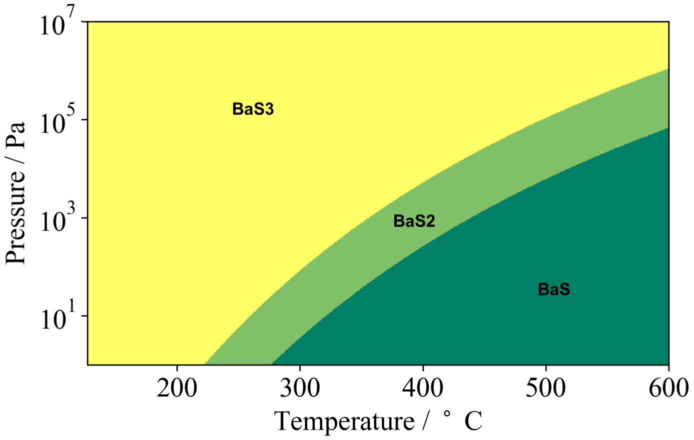

Figure S11: Most stable material in the Ba-S system displayed as a function of temperature and pressure. The material is formed in equilibrium with a single allotrope S_2. Yellow corresponds to BaS_3, mid-green corresponds to BaS_2 and dark green corresponds to BaS. The pressure is the partial pressure of S_2.

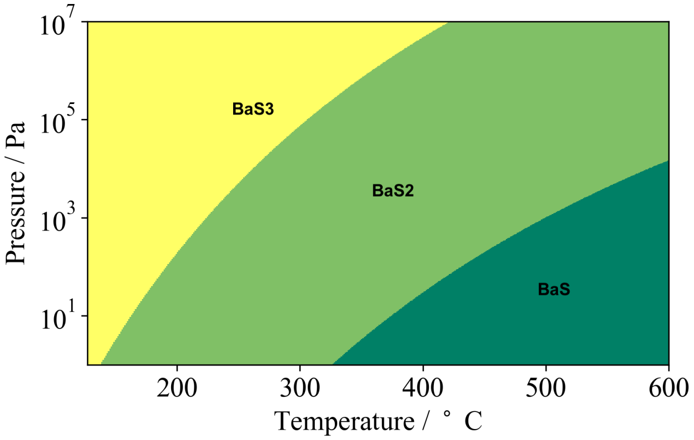

Figure S12: Most stable material in the Ba-S system displayed as a function of temperature and pressure. The material is formed in equilibrium with a single allotrope S_8. Yellow corresponds to BaS_3, mid-green corresponds to BaS_2 and dark green corresponds to BaS. The pressure is the partial pressure of S_8.

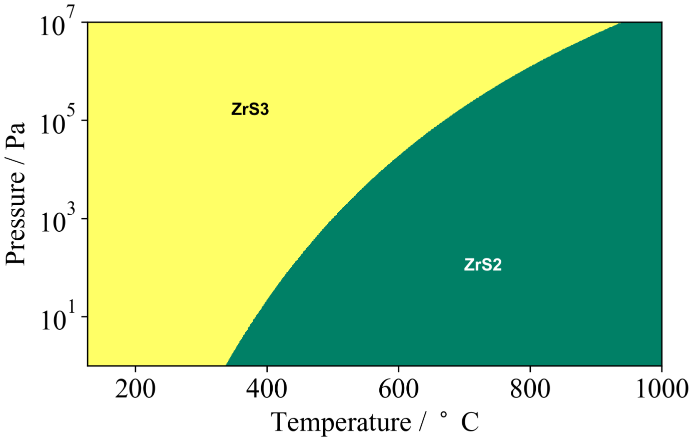

Figure S13: Most stable material in the Zr-S system displayed as a function of temperature and pressure. The material is formed in equilibrium with a single allotrope S_2. Yellow corresponds to ZrS_3 and dark-green corresponds to ZrS_2. The pressure is the partial pressure of S_2.

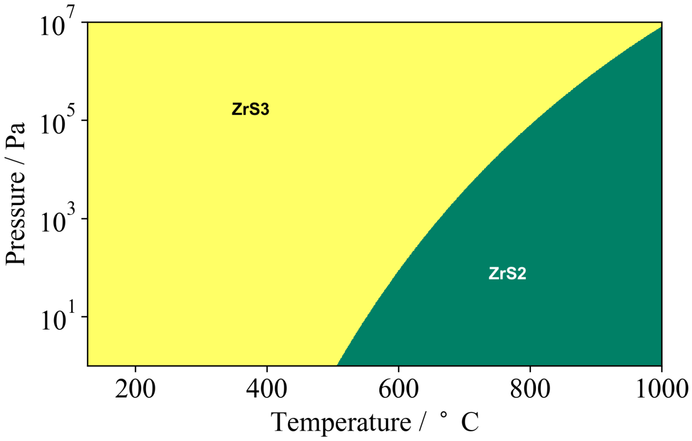

Figure S14: Most stable material in the Zr-S system displayed as a function of temperature and pressure. The material is formed in equilibrium with a single allotrope S_8. Yellow corresponds to ZrS_3 and dark-green corresponds to ZrS_2. The pressure is the partial pressure of S_8.

Extension of model to other reaction processes

There are a large number of mass-balanced reactions which are possible for the Ba-Zr-S system we are modelling. We do not present the predicted Gibbs free energy of formation for all processes, but have highlighted those of particular interest and importance. A more complete set of reactions are outlined in the Jupyter Notebook associated with this paper,[11] and we provide the first-principles data,[11,12] and software[13] required to consider any reaction for the set of Ba-Zr-S materials in this study.

We also note that it is not necessary to consider all possible product and reactant combinations; for example if we know that the reactions $A + B \longrightarrow C + D$ and $C + D \longrightarrow E$ are favourable (has a negative Gibbs free energy), we can deduce that $A + B \longrightarrow E$ will also be favourable. This reduces the number of reactions to a more manageable size.

In Figure S15 we show one result highlighted in the main text: formation of $BaZrS_3$ in an atmosphere with the single-allotrope vapour S_2, where we predict degradation at lower temperatures and higher partial pressures. For example, Figure S15 shows a thermodynamic driving force towards degradation at 1 bar and 400 °C. However this region lies above the saturation vapour pressure for sulfur, where the ideal gas model for sulfur vapour is no longer valid.

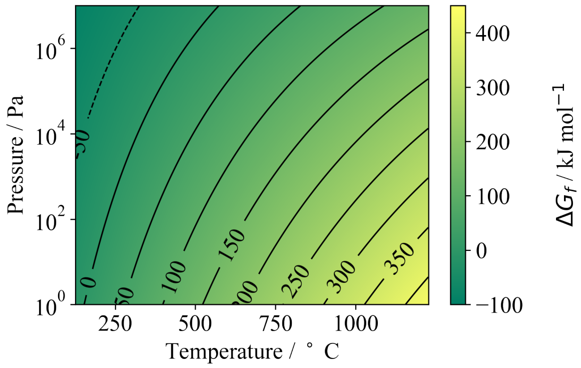

Figure S15: Gibbs free energy of the reaction $\frac{3}{2}S_2 + BaZrS_3 \rightarrow BaS_3 + ZrS_3$

Comparison of exchange-correlation functionals

Figure S16 to S29 we show results for the Gibbs free energy of formations calculated using the exchange-correlation functionals hybrid HSE06[14] and PBEsol[15] (whilst SCAN[7] is used in the main text).

In Figures S16 to S27 we see that the perovskite is predicted to remain stable with respect to elemental and binary materials, irrespective of the exchange-correlation functional used. In Figures S28 and S29 We show that the S_2/S_8 co-existance curve (where the chemical potentials of the sulfur gas allotropes on a per-atom basis are equal, $\mu_{S_8} = \mu_{S_2}$) is highly sensitive to the functional used. Compared to the PBEsol functional, the hybrid HSE06 functional predicts that S2 will dominate over a smaller region at high-temperature and low-pressure. Both disagree with a previously published higher-accuracy result using the PBE0 functional,[8] which shows S_2 will dominate in the temperature and pressure conditions typically used for $BaZrS_3$ synthesis (further discussion on this is provided in the Methods section of the main text).

All of the reactions considered in the main text can be analysed using the HSE06 or PBEsol functionals using the dataset,[11,12] workflows[11] and software[13] accompanying this paper.

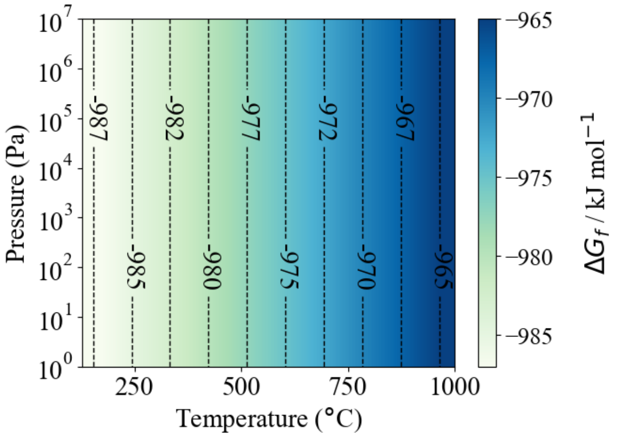

Figure S16: Gibbs free energy of Ba + Zr + 3S(s) → $BaZrS_3$, calculated using the hybrid HSE06 exchange-correlation functional.

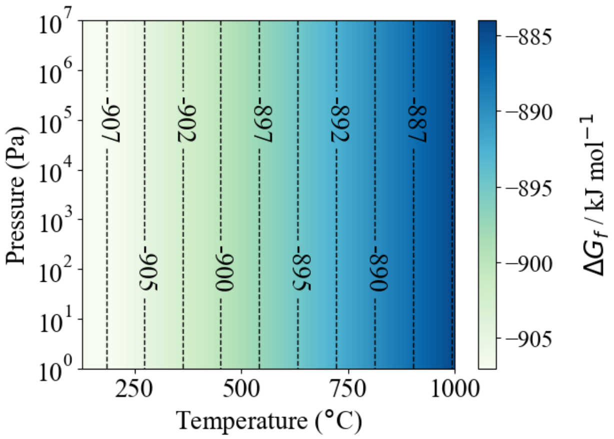

Figure S17: Gibbs free energy of Ba + Zr + 3S(s) $\rightarrow$ $BaZrS_3$, calculated using the PBEsol exchange-correlation functional.

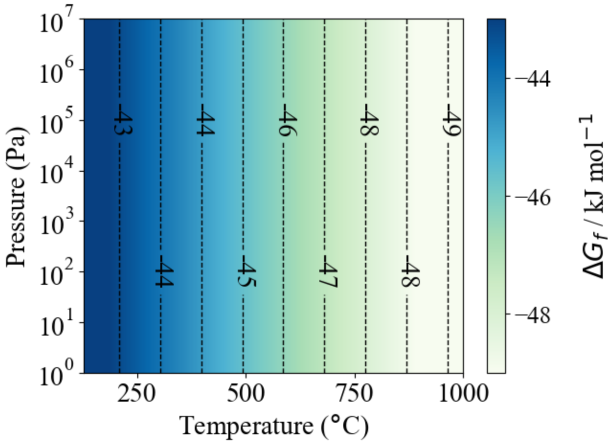

Figure S18: Gibbs free energy of BaS + ZrS_2 $\rightarrow$ $BaZrS_3$, calculated using the hybrid HSE06 exchange-correlation functional.

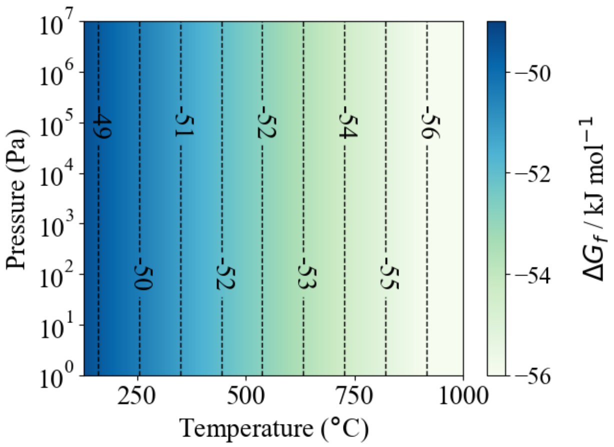

Figure S19: Gibbs free energy of BaS + ZrS_2 → $BaZrS_3$, calculated using the PBEsol exchange-correlation functional.

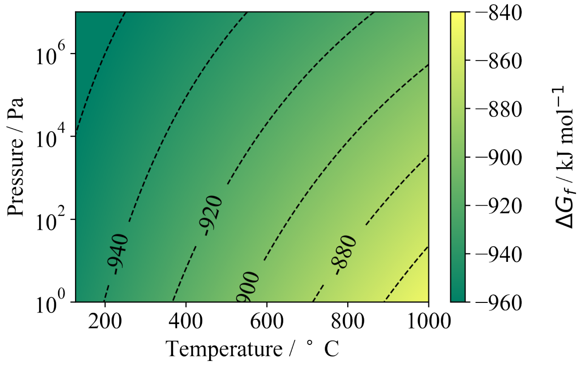

Figure S20: Gibbs free energy of Ba + Zr + $\frac{3}{8}S_8(g)$ → $BaZrS_3$, calculated using the hybrid HSE06 exchange-correlation functional.

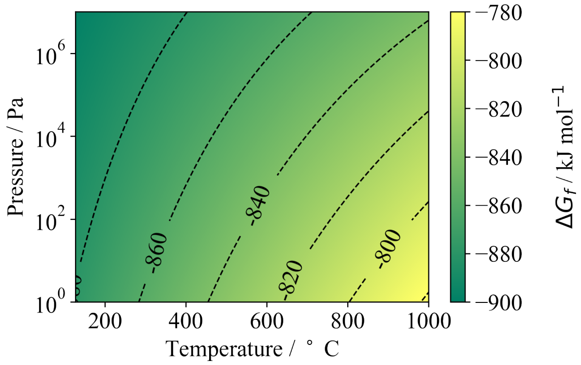

Figure S21: Gibbs free energy of Ba + Zr + $\frac{3}{8}S_8(g)$ → $BaZrS_3$, calculated using the PBEsol exchange-correlation functional.

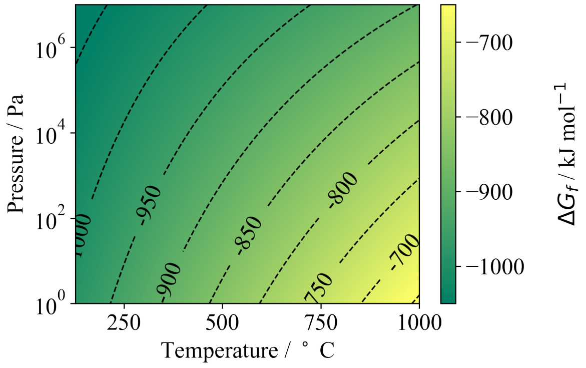

Figure S22: Gibbs free energy of Ba + Zr + $\frac{3}{2}S_2(g) \rightarrow BaZrS_3$, calculated using the hybrid HSE06 exchange-correlation functional.

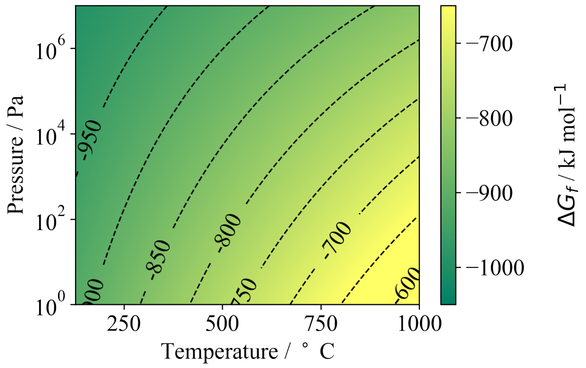

Figure S23: Gibbs free energy of Ba + Zr + $\frac{3}{2}S_2(g) \rightarrow BaZrS_3$, calculated using the PBEsol exchange-correlation functional.

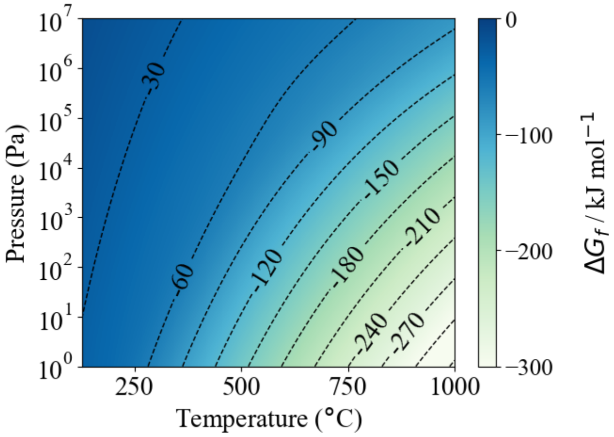

Figure S24: Gibbs free energy of $BaZrS_3 + 3S_{mix}(g) \rightarrow ZrS_3 + BaS_3$, calculated using the hybrid HSE06 exchange-correlation functional.

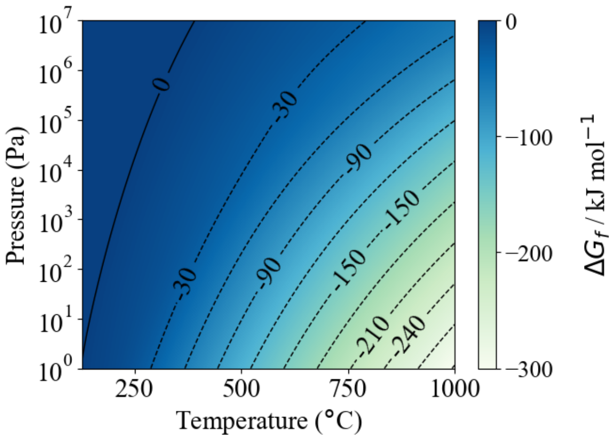

Figure S25: Gibbs free energy of $BaZrS_3$ + $3S_{mix}(g) \rightarrow ZrS_3 + BaS_3$, calculated using the PBEsol exchange-correlation functional.

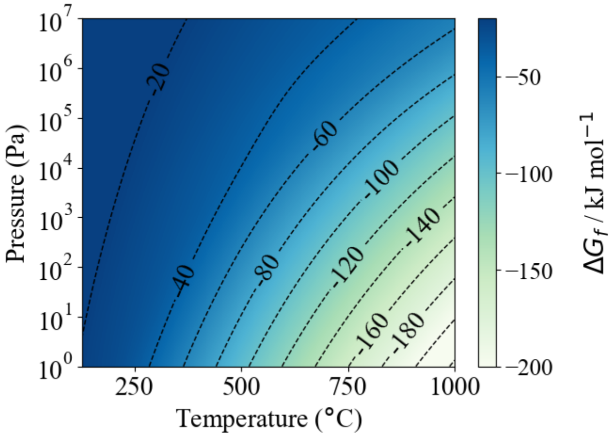

Figure S26: $BaZrS_3 + 2S_{mix}(g) \rightarrow ZrS_3 + BaS_2$, calculated using the hybrid HSE06 exchange-correlation functional.

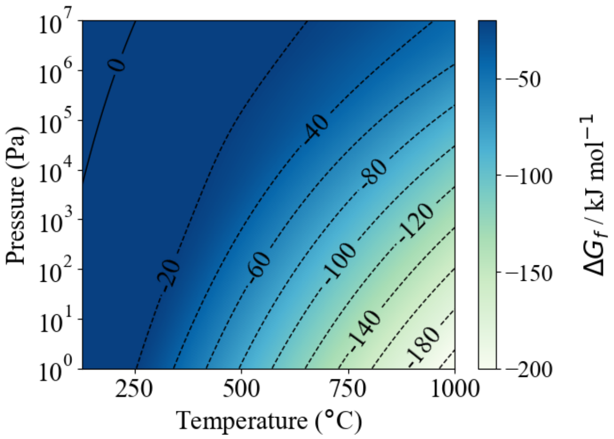

Figure S27: $BaZrS_3$ + $2S_{mix}(g)$ $\rightarrow$ ZrS_3 + BaS_2, calculated using the PBEsol exchange-correlation functional.

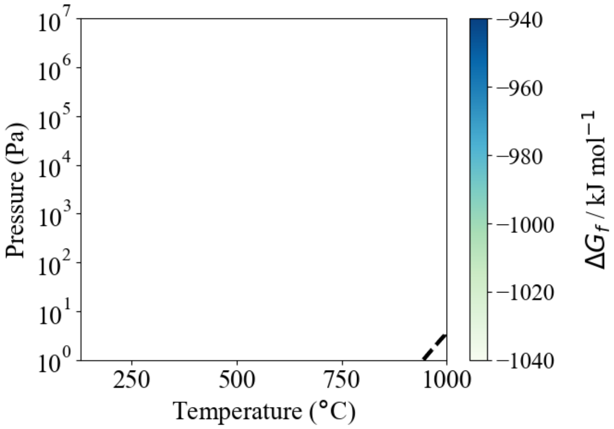

Figure S28: The coexistance curve for single allotrope S_2 vapour and single allotrope S_8 vapour, with total energies calculated using the hybrid HSE06 exchange-correlation functional. The dashed line indicates where the chemical potentials of the sulfur gas allotropes on a per-atom basis are equal, $\mu_{S8} = \mu_{S2}$. S_8 dominates in the top left region, S2 dominates in the bottom right.

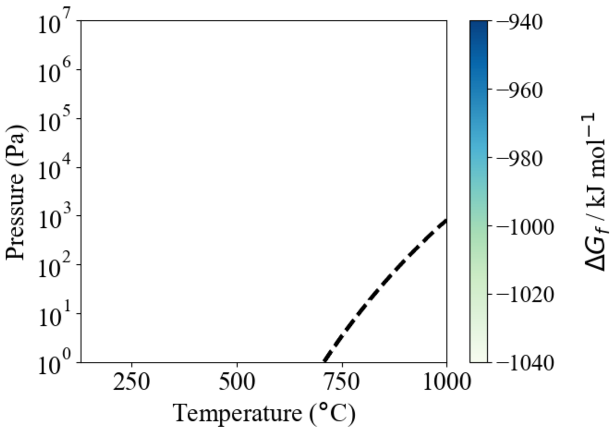

Figure S29: The coexistance curve for single allotrope S_2 vapour and single allotrope S_8 vapour, with total energies calculated using the PBEsol exchange-correlation functional. The dashed line indicates where the chemical potentials of the sulfur gas allotropes on a per-atom basis are equal, $\mu_{S8} = \mu_{S2}$. S_8 dominates in the top left region, S2 dominates in the bottom right.

Electronic bandstructures

Figures S30 to S36 show the electronic bandstructures for $BaZrS_3$ and the binary materials selected in this study, calculated using the HSE06 functional. The bandstructures are generated from density functional theory calculations, as outlined in the methods section of the main text. They have been plotted using `aimstools` (github.com/romankempt/aimstools).

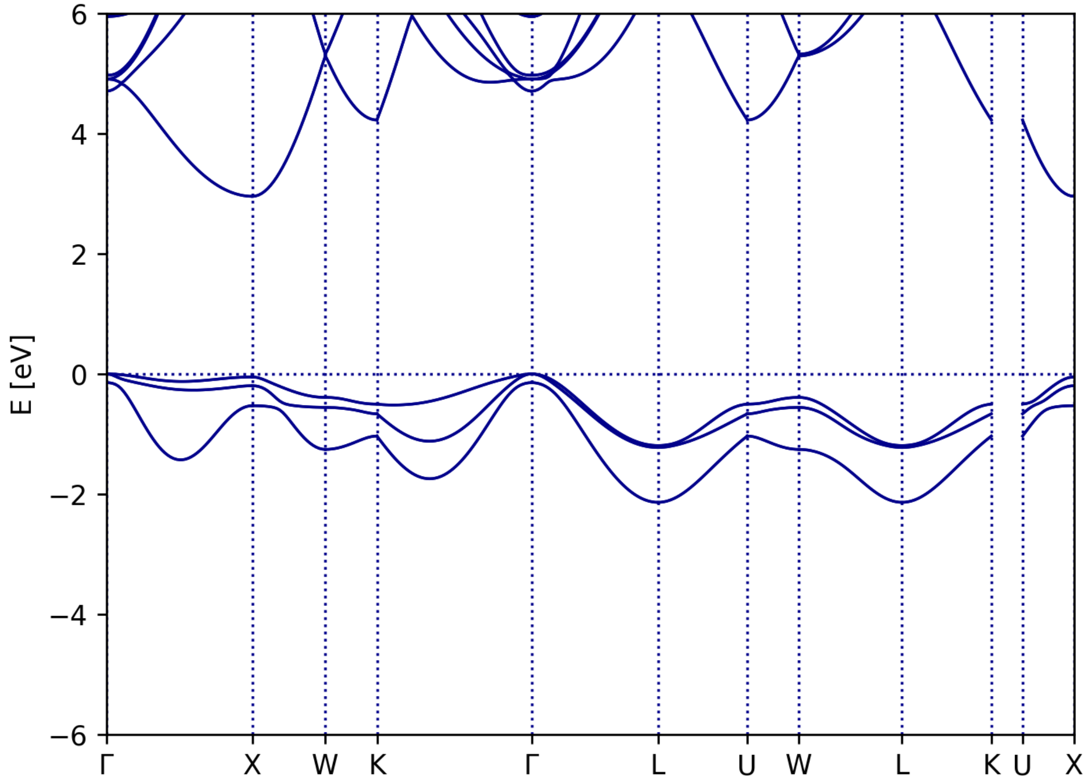

Figure S30: BaS ($Fm\bar{3}m$) electronic band structure

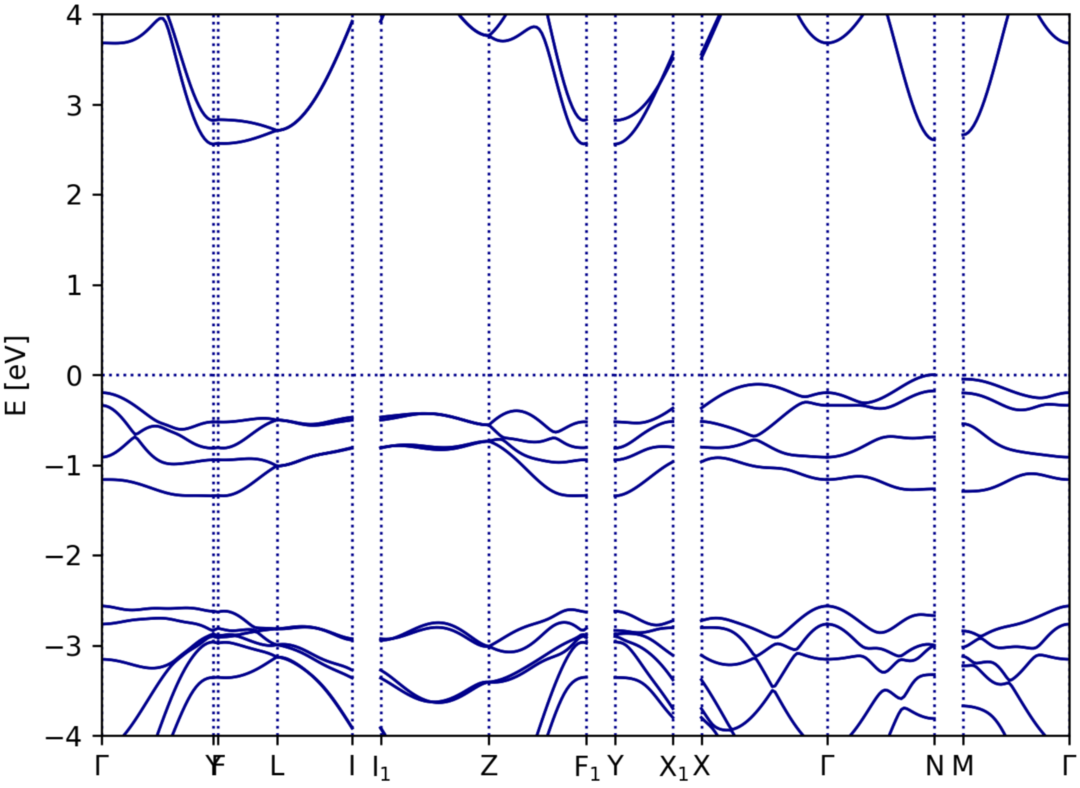

Figure S31: BaS_2 (C2/c) electronic band structure

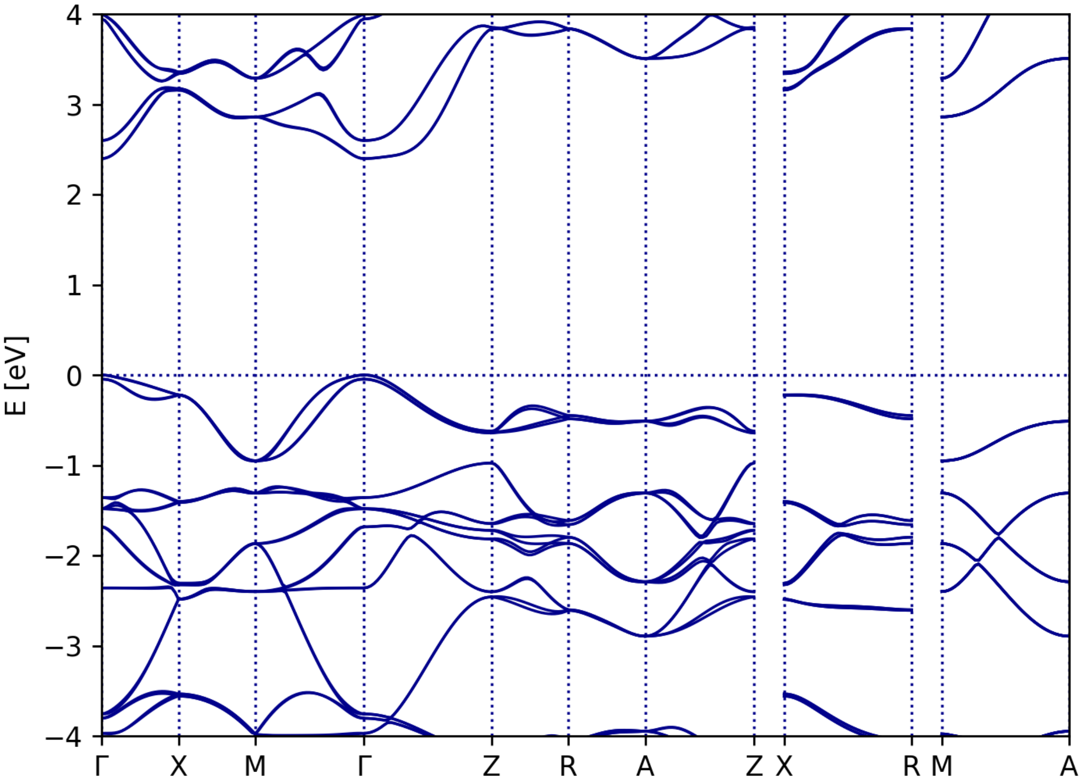

Figure S32: BaS_3 ($P\bar{4}2_1m$) electronic band structure

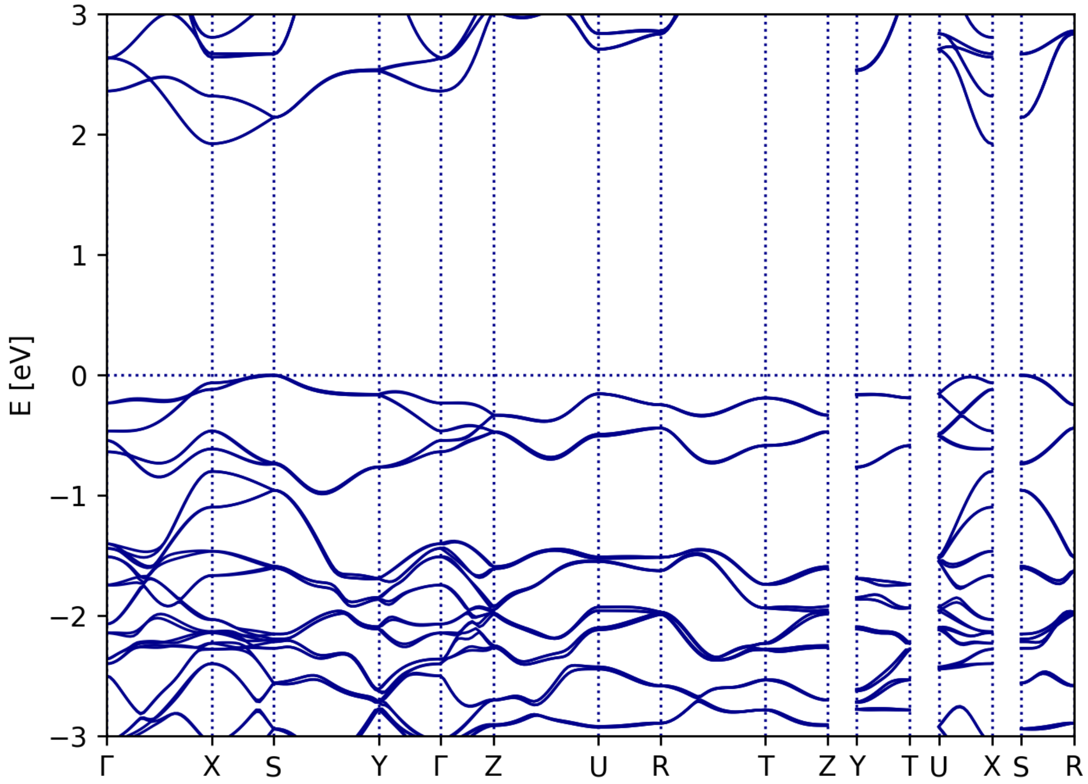

Figure S33: BaS_3 ($P2_12_12$) electronic band structure

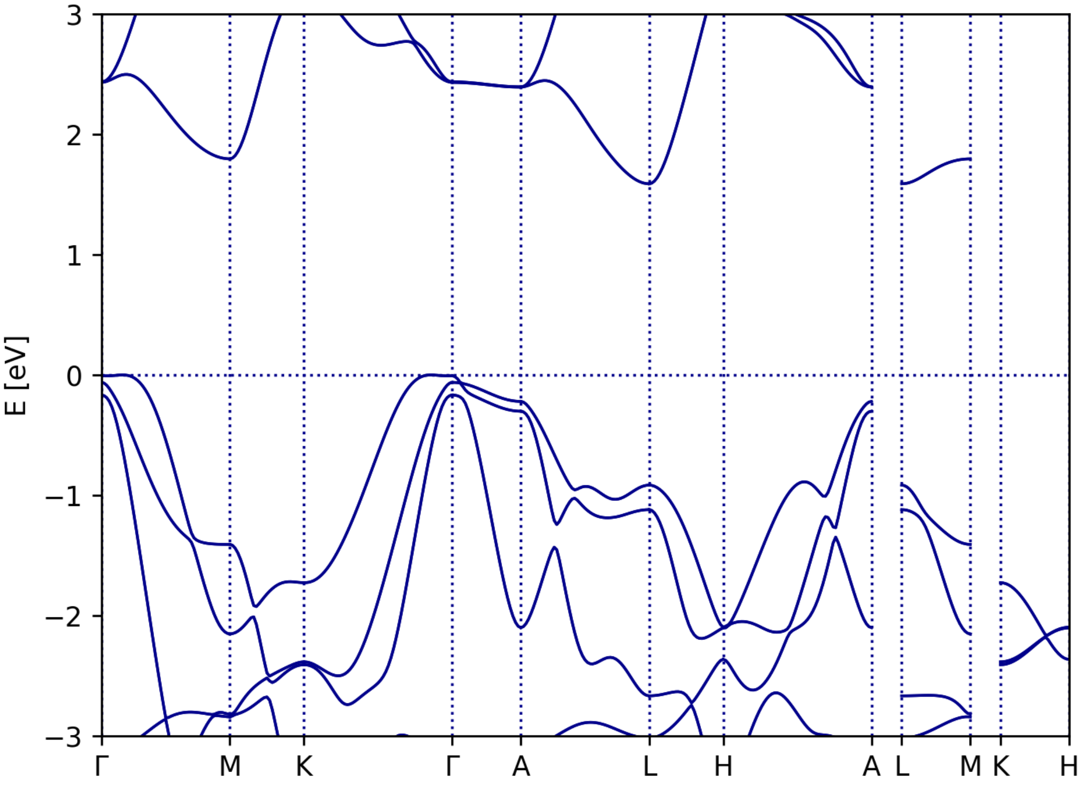

Figure S34: ZrS_2 ($P\bar{3}m1$) electronic band structure

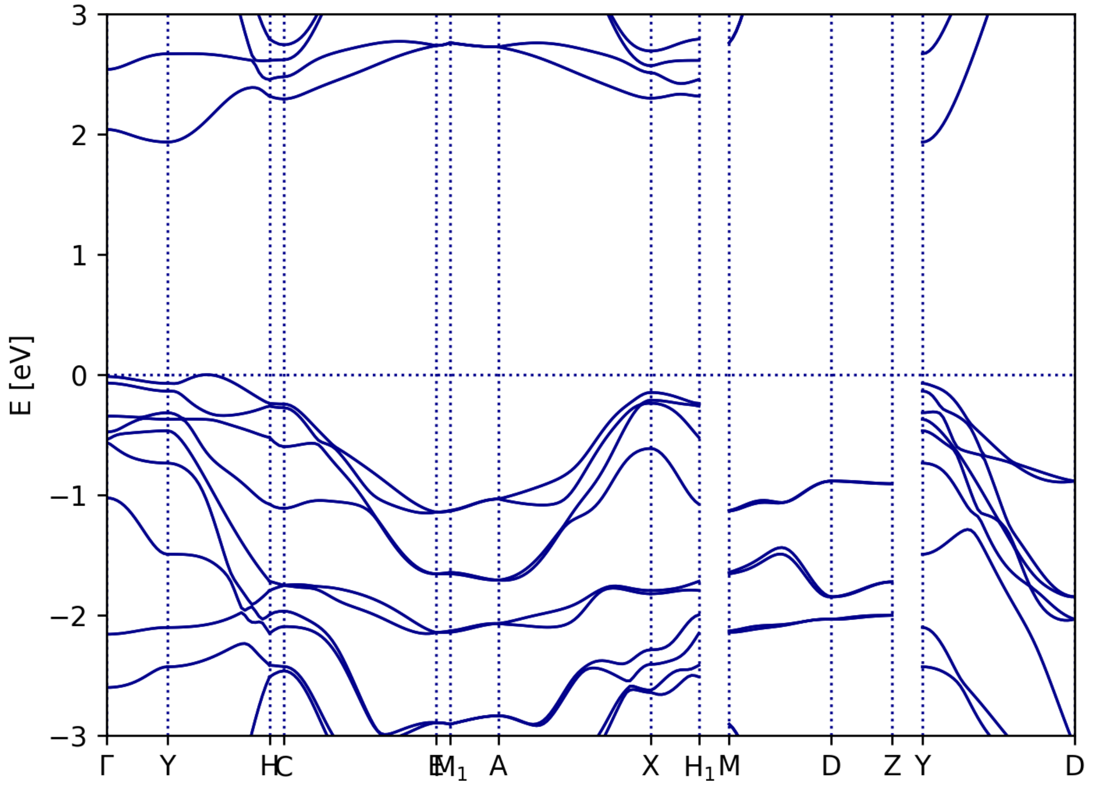

Figure S35: ZrS_3 ($P2_1/m$) electronic band structure

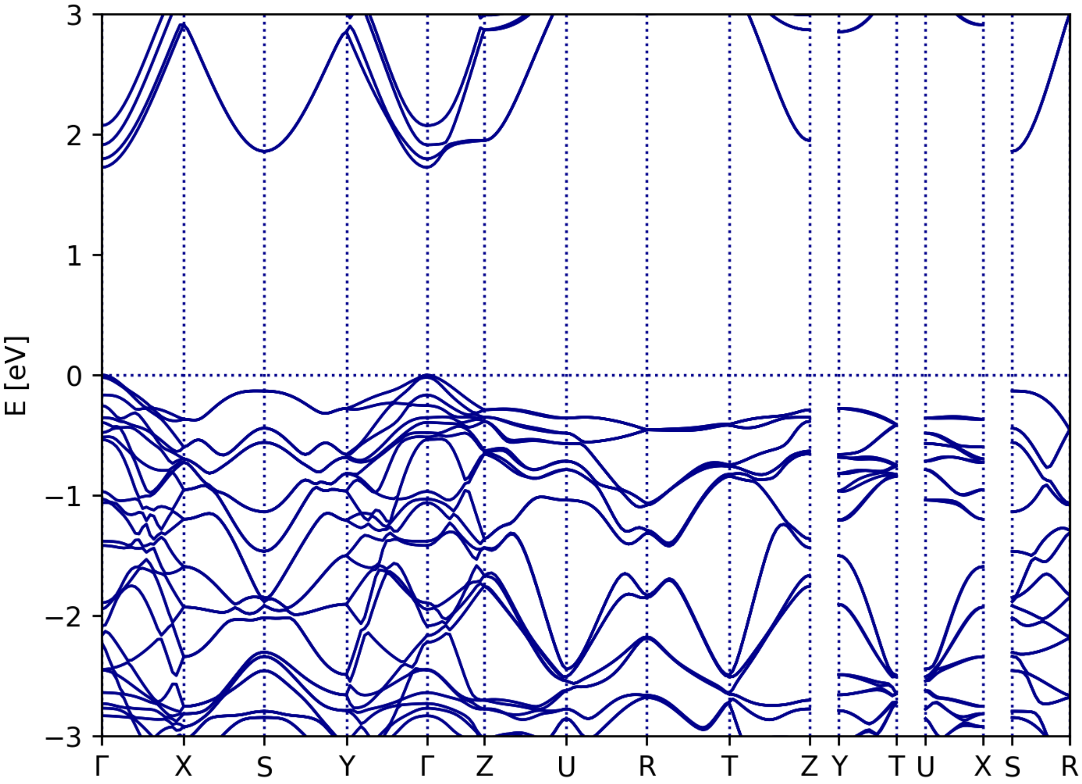

Figure S36: $BaZrS_3$ ($Pnma$) electronic band structure

Phonon bandstructures

Figures S37 to S45 present the phonon bandstructures for $BaZrS_3$ and all of the binary materials selected for this study. The bandstructures are generated using first-principles lattice dynamics, as outlined in the methods section of the main text. They have been plotted using `sumo`.[16] The only materials with a kinetic instability (ie, which have phonon mode(s) with an imaginary frequency) are the two ZrS compounds. However we find that these are also thermodynamically unstable across the full temperature and pressure range considered in this study.

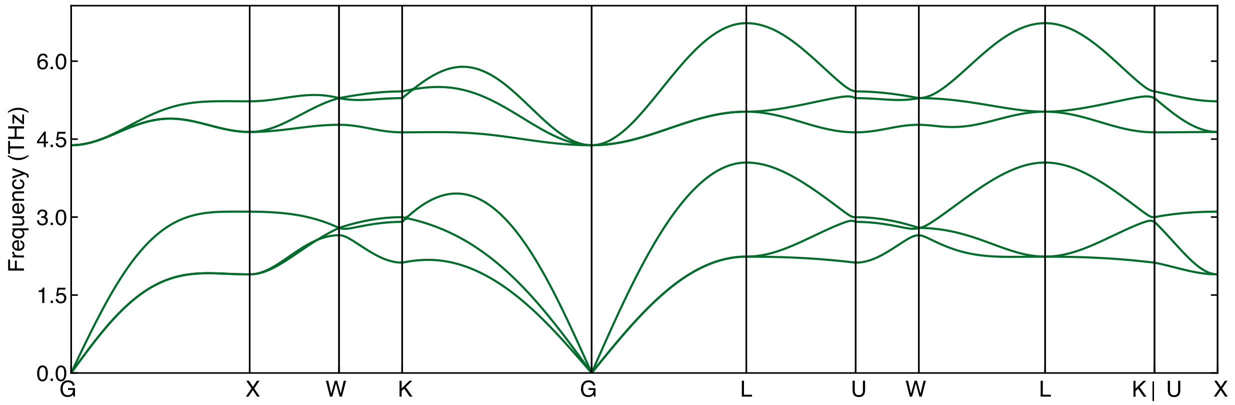

Figure S37: BaS ($Fm\bar{3}m$) phonon band structure

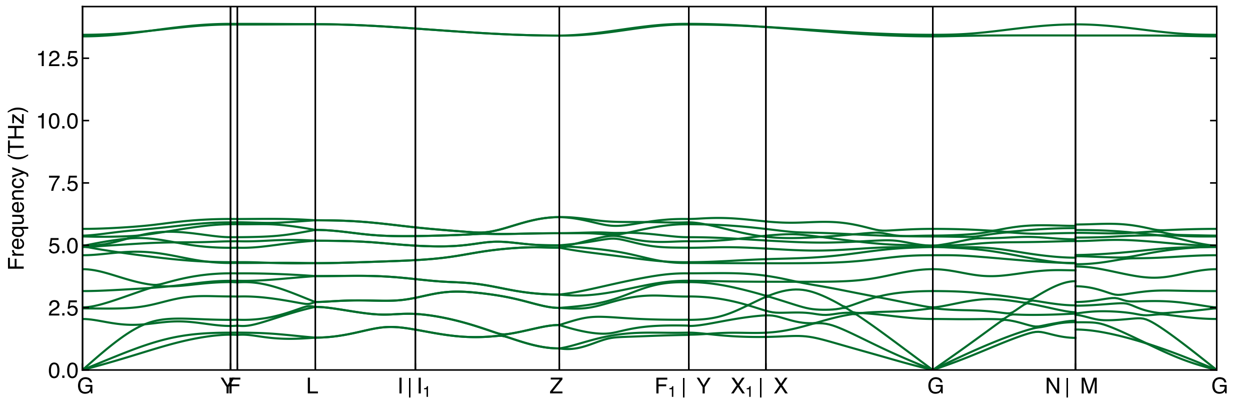

Figure S38: BaS_2 ($C2/c$) phonon band structure

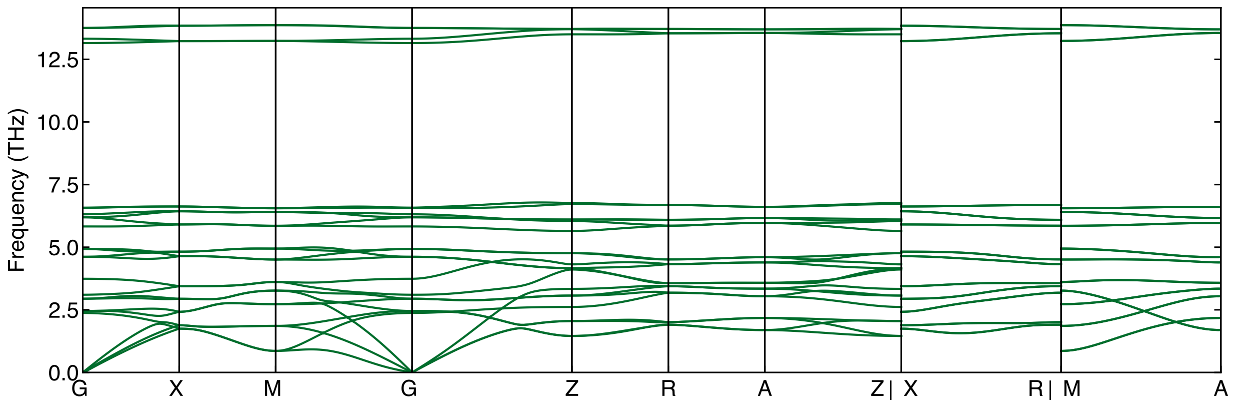

Figure S39: BaS_3 ($P\bar{4}2_1m$) phonon band structure

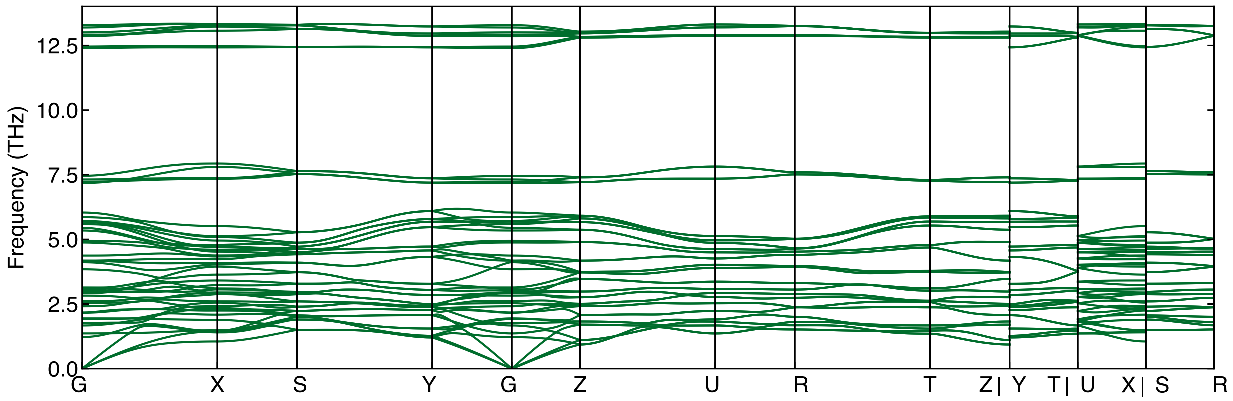

Figure S40: BaS_3 ($P2_12_12$) phonon band structure

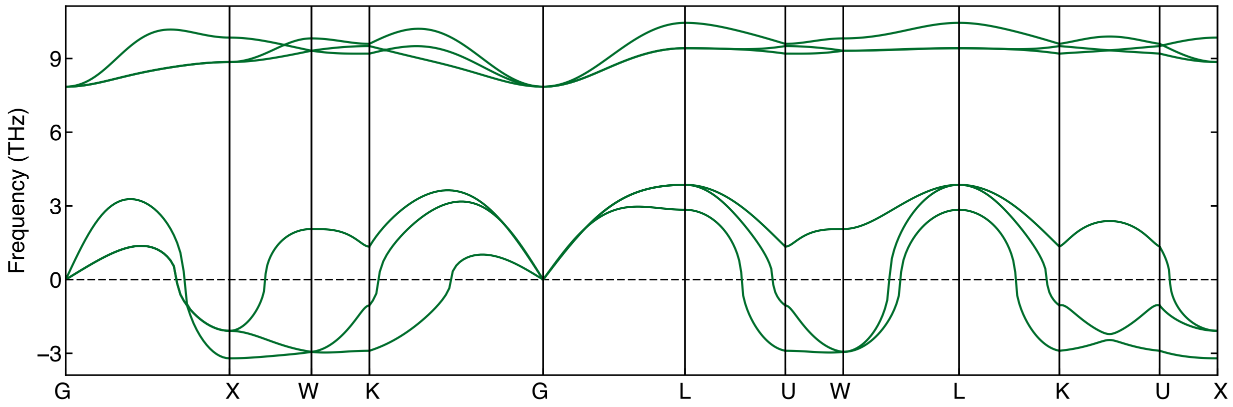

Figure S41: ZrS ($Fm\bar{3}$m) phonon band structure

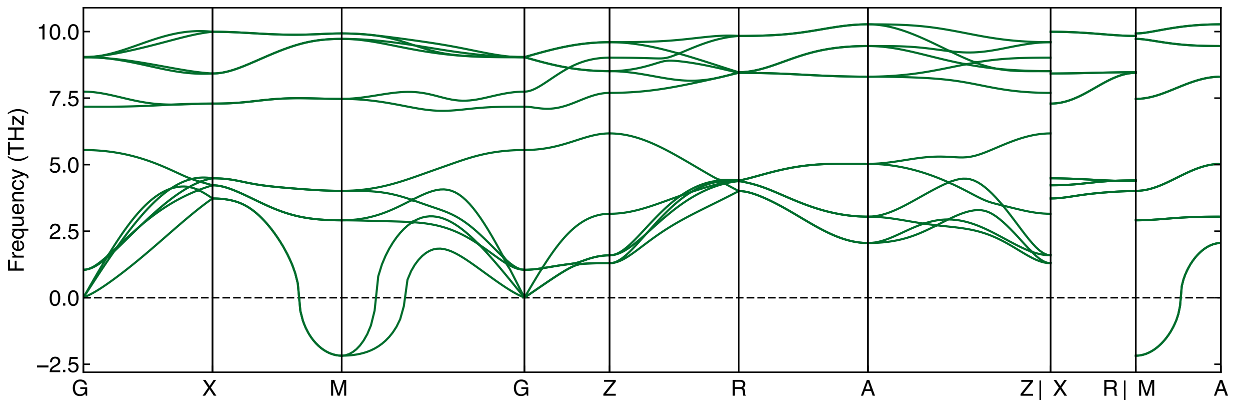

Figure S42: ZrS ($P4/nmm$) phonon band structure

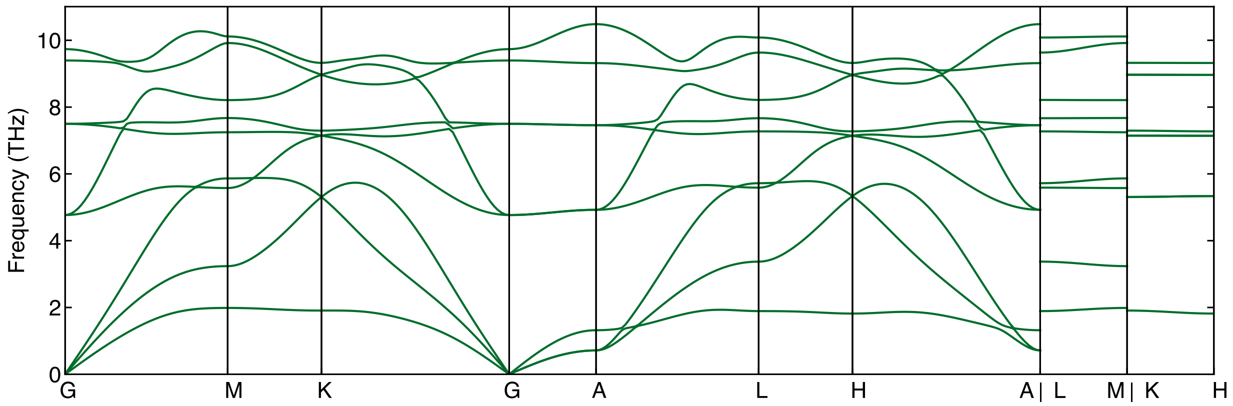

Figure S43: ZrS_2 ($P\bar{3}m1$) phonon band structure

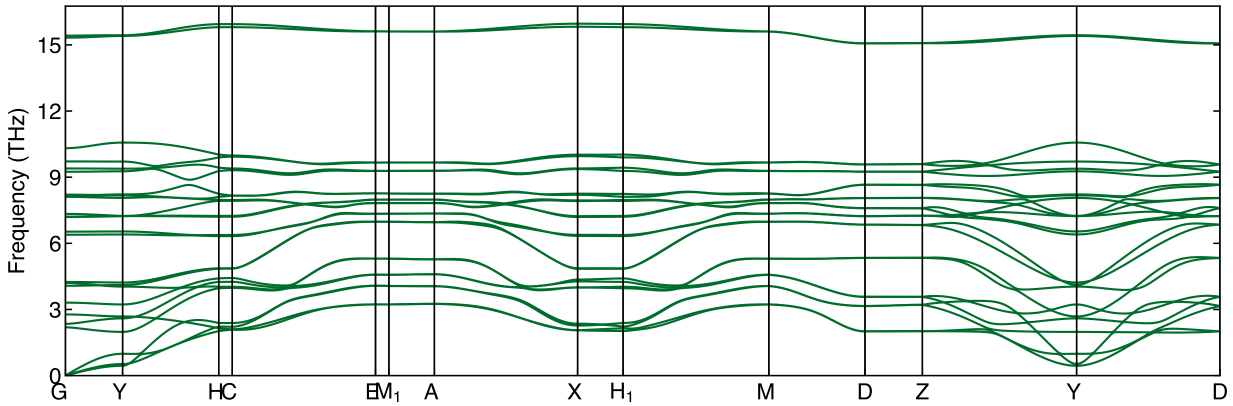

Figure S44: ZrS_3 ($P2_1/m$) phonon band structure

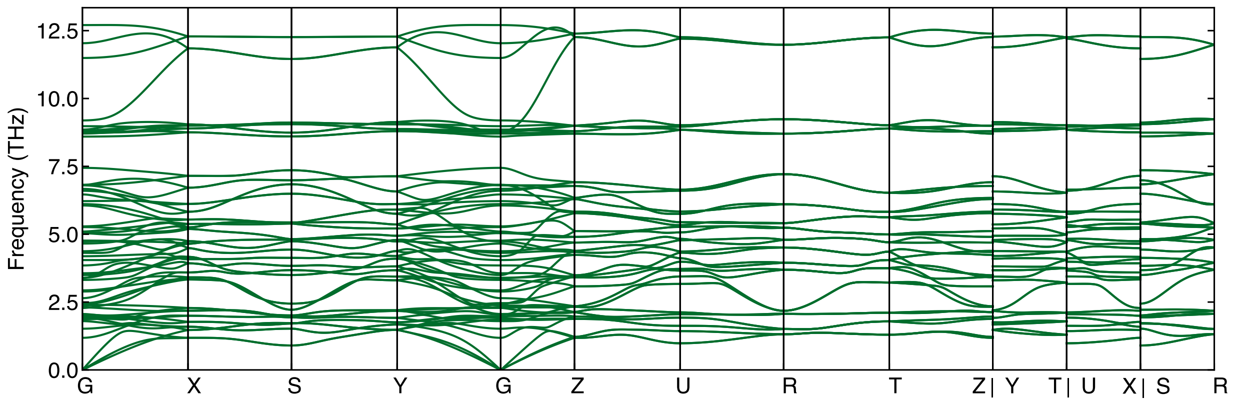

Figure S45: $BaZrS_3$ ($Pnma$) phonon band structure

Formation of Ruddlesden-Popper phases

As outlined in the main text, Ruddlesden-Popper (RP) phases are reported to form at high temperatures and using the same methodology as outlined in this paper we have demonstrated that they are energetically accessible above 1000 K.[3] An alternative RP formation mechanism is driven by a Zr or S deficit during synthesis. This will reduce the respective chemical potentials, allowing formation of the Zr- and S- deficit RP phases. By the same argument, the formation of RP-phases will be less energetically favourable when there is a sulfur-rich environment. Ruddlesden-Popper phases are not examined here as we focus our attention on perovskite formation at moderate temperatures and with an excess of sulfur.

Ba–Zr–S ternary phase diagrams

In Figures S46 and S47 we show the phase diagram for the Ba-Zr-S system at 0K and 800K respectively. The phase diagrams have been calculated using ThermoPot[13] and Pymatgen.[6] Total energies (using the SCAN exchange-correlation functional) and vibrational contributions have been calculated as outlined in the Methods section of the main text.

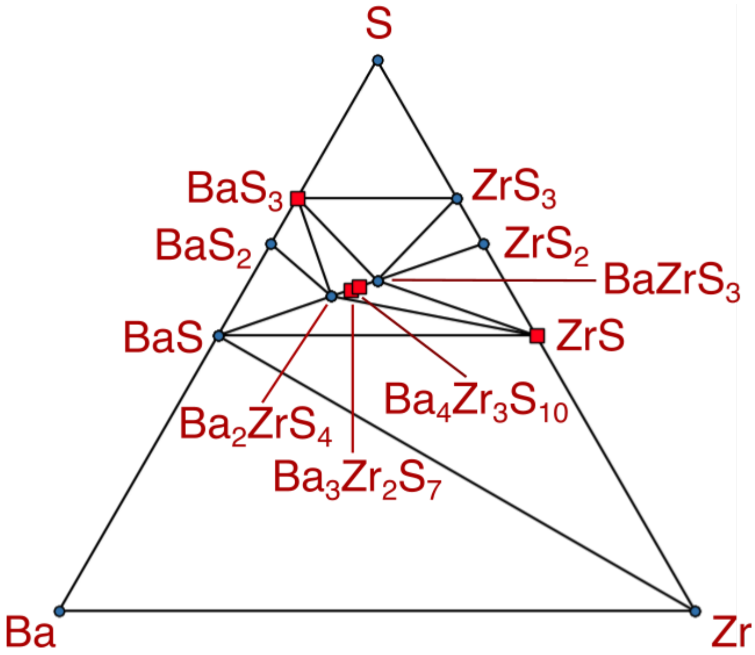

Figure S46: Phase diagram for the Ba-Zr-S system at 0K. The blue points indicate stable phases on the convex hull, whilst the red squares indicate an unstable phase lying within 0.2 eV/atom of the convex hull. Unstable phases above this cutoff are not displayed.

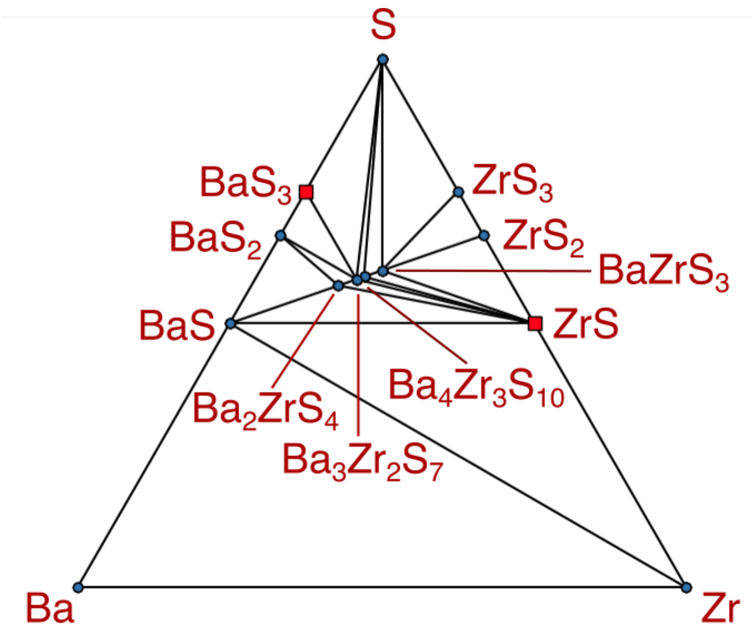

Figure S47: Phase diagram for the Ba-Zr-S system at 800K. The blue points indicate stable phases on the convex hull, whilst the red squares indicate an unstable phase lying within 0.2 eV/atom of the convex hull. Unstable phases above this cutoff are not displayed.

References

(1) Jain, A.; Ong, S. P.; Hautier, G.; Chen, W.; Richards, W. D.; Dacek, S.; Cholia, S.; Gunter, D.; Skinner, D.; Ceder, G.; Persson, K. A. Commentary: The Materials Project: A materials genome approach to accelerating materials innovation. *APL Materials* **2013**, *1*, 011002.

(2) Zagorac, D.; Müller, H.; Ruehl, S.; Zagorac, J.; Rehme, S. Recent developments in the Inorganic Crystal Structure Database: theoretical crystal structure data and related features. *Journal of applied crystallography* **2019**, *52*, 918–925.

(3) Kayastha, P.; Tiwari, D.; Holland, A.; Hutter, O. S.; Durose, K.; Whalley, L. D.; Longo, G. High-temperature equilibrium of 3D and 2D chalcogenide perovskites. *Solar RRL* **2023**, 2201078.

(4) Sun, W.; Dacek, S. T.; Ong, S. P.; Hautier, G.; Jain, A.; Richards, W. D.; Gamst, A. C.; Persson, K. A.; Ceder, G. The thermodynamic scale of inorganic crystalline metastability. *Science Advances* **2016**, *2*, e1600225.

(5) Aykol, M.; Dwaraknath, S. S.; Sun, W.; Persson, K. A. Thermodynamic limit for synthesis of metastable inorganic materials. *Science Advances* **2018**, *4*, eaaq0148.

(6) Ong, S. P.; Wang, L.; Kang, B.; Ceder, G. Li-Fe-P-O_2 Phase Diagram from First Principles Calculations. *Chemistry of Materials* **2008**, *20*, 1798–1807.

(7) Sun, J.; Ruzsinszky, A.; Perdew, J. P. Strongly constrained and appropriately normed semilocal density functional. *Physical review letters* **2015**, *115*, 036402.

(8) Jackson, A. J.; Tiana, D.; Walsh, A. A universal chemical potential for sulfur vapours. *Chemical Science* **2016**, *7*, 1082–1092.

(9) Adamo, C.; Barone, V. Toward reliable density functional methods without adjustable parameters: The PBE0 model. *The Journal of Chemical Physics* **1999**, *110*, 6158–6170.

(10) Yang, R.; Nelson, J.; Fai, C.; Yetkin, H. A.; Werner, C.; Tervil, M.; Jess, A. D.; Dale, P. J.; Hages, C. J. A low-temperature growth mechanism for chalcogenide perovskites. *Chemistry of Materials* **2023**, *35*, 4743–4750.

(11) Kayasatha, P.; Whalley, L. D. 2023-BaZrS3-Thermodynamic-Model. Zenodo repository, 2023; `https://dx.doi.org/10.5281/zenodo.10424511`.

(12) Kayastha, P.; Whalley, L. D. Ba-Zr-S dataset. NOMAD repository, 2023; `https://dx.doi.org/10.17172/NOMAD/2023.12.21-1`.

(13) Kayastha, P.; Jackson, A. J.; Walsh, A.; Whalley, L. D. ThermoPot software. Zenodo repository, 2023; `https://dx.doi.org/10.5281/zenodo.10412986`.

(14) Krukau, A. V.; Vydrov, O. A.; Izmaylov, A. F.; Scuseria, G. E. Influence of the exchange screening parameter on the performance of screened hybrid functionals. *The Journal of chemical physics* **2006**, *125*, 224106.

(15) Perdew, J. P.; Ruzsinszky, A.; Csonka, G. I.; Vydrov, O. A.; Scuseria, G. E.; Constantin, L. A.; Zhou, X.; Burke, K. Restoring the density-gradient expansion for exchange in solids and surfaces. *Physical review letters* **2008**, *100*, 136406.

(16) Ganose, A. M.; Jackson, A. J.; Scanlon, D. O. sumo: Command-line tools for plotting and analysis of periodic ab-initio calculations. *Journal of Open Source Software* **2018**, *3*, 717.
